# Intracellular trafficking SNARE protein, syntaxin-6, modifies prion cellular phenotypes and risk of disease development in vivo

**DOI:** 10.1007/s00401-025-02946-8

**Published:** 2025-11-04

**Authors:** Elizabeth Hill, Mitali M. Patel, Juan M. Ribes, Jacqueline Linehan, Fuquan Zhang, Tatiana Jakubcova, Shyma Hamdan, Andrew Tomlinson, Tiziana Ercolani, Christian Schmidt, Parvin Ahmed, George Thirlway, Fabio Argentina, Aline T. Marinho, Emma Jones, Nicholas Kaye, Craig Fitzhugh, Graham S. Jackson, Sebastian Brandner, Peter-Christian Klöhn, John Collinge, Thomas J. Cunningham, Simon Mead

**Affiliations:** 1 MRC Prion Unit at UCL, UCL Institute of Prion Diseases, Courtauld Building, 33 Cleveland Street, London, W1W 7FF UK; 2 School of Biochemistry, Biomedical Sciences Building, University of Bristol, Bristol, BS8 1TD UK; 3 Department of Neurodegenerative Disease, UCL Queen Square Institute of Neurology, London, WC1N 3BG UK

**Keywords:** Prion disease, Prion-like mechanisms, Neurodegeneration, Functional genetics, Creutzfeldt–Jakob disease

## Abstract

Increased expression of syntaxin-6, a SNARE protein involved in intracellular protein trafficking, is a proposed genetic risk mechanism for sporadic prion disease and progressive supranuclear palsy, as well as being implicated in Alzheimer’s disease. However, no study has validated its functional role in prion disease, its mechanism of action nor explored the disease stage at which it is acting. Here, we show that syntaxin-6 knockdown in cellular models increases cell-associated infectivity, whilst overexpression produces the opposite effect. This observation is broadly consistent across multiple cell types and prion strains. Furthermore, syntaxin-6 knockdown leads to an accumulation of perinuclear disease-related PrP, consistent with a trafficking mechanism, and alters the morphology of disease-related PrP. We demonstrate that syntaxin-6 knockdown reduces the secretion of prions from infected cells, which provides a mechanism for the prion-related cellular phenotypes observed. Complementary in vivo studies showed that syntaxin-6 influences early stages of prion disease in experimental mice, increasing transmission risk after inoculation with low prion doses. Conversely, syntaxin-6 does not affect prion propagation kinetics or toxicity during established disease. Taken together, our studies firmly establish syntaxin-6 as a modifier of prion pathogenesis with a role in prion trafficking and export. Our findings further suggest that syntaxin-6 modifies the risk of the establishment of disease in line with its genetic association in humans. Thus, this work provides important insights into the role of a pleiotropic prion/prion-like modifier, grounded in human genetics evidence, which may have wider relevance to other neurodegenerative diseases.

**Supplementary Information:**

The online version contains supplementary material available at 10.1007/s00401-025-02946-8.

## Background

Prion diseases are fatal, infectious neurodegenerative diseases, characterised by recruitment of the cellular prion protein (PrP^C^) into fibrillar, multimeric amyloid assemblies [12]. Sporadic Creutzfeldt–Jakob disease (sCJD), the most common human prion disease, is thought to occur when seeds of misfolded PrP form spontaneously and then propagate by fibril growth, fission and cell-to-cell spread. Prions also have distinct and transmissible clinicopathological variants, known as strains, which are likely encoded in PrP fibril conformation [13]. Decades of research indicate that other neurodegenerative disorders also involve ‘prion-like’ mechanisms [12, 25].

Human genetics studies offer a valuable approach to identify molecular players implicitly causal in disease pathogenesis. Variants at the syntaxin-6 (*STX6)* locus have been identified by genome-wide association studies (GWAS) as shared genetic risk factors for sCJD [24] and the most common primary tauopathy, progressive supranuclear palsy (PSP) [9, 10, 15, 16, 19]. Furthermore, a recent proteome-wide association study (PWAS) found a causal association between STX6 protein levels and AD [50]. These studies propose syntaxin-6 as a candidate prion/prion-like modifier, exerting pleiotropic risk effects across multiple neurodegenerative diseases. However, genetic data only provide suggestive evidence for the most likely gene driving disease risk at a locus. Although increased *STX6* expression is linked to disease risk by transcriptomic and proteomic analyses [15, 22, 27, 50], providing a causal genetic mechanism, functional studies are necessary to validate this mechanism and translate these genetic discoveries into more precise pathogenic disease mechanisms.

Syntaxin-6 encodes an intracellular trafficking protein [5, 6], which functions in a highly cell-type specific manner [49]. Recent work suggests that *STX6* risk variant-driven expression changes are strongest in oligodendrocytes [7, 15, 22, 27], supporting its study in a multicellular system to capture non-cell autonomous effects. Facilitating this, we generated *Stx6*^*−/−*^ mice in collaboration with MRC Harwell, which are viable with no gross neurological, physiological or behavioural phenotypes [23]. However, prion infection of *Stx6*^*−/−*^ mice only had very modest effects on the endpoint clinical outcome measures providing no clear evidence of a modifying role [23].

Prion disease proceeds in two distinct mechanistic phases in prion-inoculated mice [43, 44]. The clinically and neuropathologically silent first phase is characterised by an exponential increase in prion titre. This is followed by a second phase, after prion titres have plateaued, where neurotoxicity and neuropathological features become established, culminating in the onset of clinical disease. A similar sequence of events may occur in sCJD, although in this case, disease begins with rare spontaneous prion formation, which then spreads throughout the central nervous system. We hypothesised that syntaxin-6 confers risk of prion disease by modification of one of these key three stages: the establishment of disease, prion propagation or prion-induced toxicity.

The main aims of this work were to functionally validate a role for syntaxin-6 in prion pathogenesis*,* inform on causal pathogenic disease mechanisms, and explore the disease stage at which syntaxin-6 is acting. To achieve this, we performed comprehensive cellular studies which supported a role for syntaxin-6 in the trafficking and export of prions. Additionally, we conducted prion transmission studies in *Stx6*^+*/*+^ and *Stx6*^*−/−*^ mice which supported syntaxin-6 as a modifier of prion pathogenesis in vivo, modulating early stages of the disease, with no discernable effect during established disease. Therefore, this work validates a role for syntaxin-6 in prion pathogenesis in both cellular and mouse models, thus providing important insights into the role of a pleiotropic prion/prion-like modifier, grounded in human genetics evidence.

## Materials and methods

### Ethics and research governance

All experimental procedures employing mouse-adapted prions were conducted in microbiological containment level 2 (CL2) or level 3 (CL3) facilities with strict adherence to safety protocols and guidelines. Work with mice was performed under approval and licence granted by the UK Home Office [Animals (Scientific Procedures) Act 1986], which conforms to UCL institutional guidelines and Animal Research: Reporting of In Vivo Experiments (ARRIVE) guidelines (www.nc3rs.org.uk/ARRIVE/). Full ethical review and research governance details are included in the Supplementary Methods (supplementary file 1).

### Cell-based assays

Cell lines with stable manipulation of syntaxin-6 were generated using the PiggyBac transposon system or via stable transfection as detailed in the Supplementary Methods (supplementary file 1). Transient *Stx6* knockdown was achieved by reverse transfection with pools of 30 custom-designed siRNAs (siPools). Validation of gene expression manipulation was confirmed by western blotting. Infectivity titres were measured using the scrapie cell assay (SCA) and the automated SCA (ASCA) platforms with PrP levels quantified by flow cytometry, RT-qPCR, and western blotting, as well as confocal microscopy being used to assess for altered distribution of disease-associated PrP. The cycloheximide assay was used to assess PrP degradation kinetics. Cell viability was assessed using the CellTitre-Glo assay. All cell culture procedures, including transfection protocols and assay conditions, are detailed in the Supplementary Methods (supplementary file 1).

### Animal studies

*Stx6*^+*/*+^ and *Stx6*^*−/−*^ mice were bred on a C57BL/6N background and housed in pathogen-free, individually ventilated cages under standard environmental conditions (20–24 °C, 45–65% humidity, 12/12-h light/dark cycle). They were provided with irradiated feed and reverse osmosis water ad libitum. Genotyping was performed using ear biopsies by polymerase chain reaction (PCR) as described in the Supplementary Methods (supplementary file 1). The health of the mice was closely monitored throughout the study. Further details on the breeding strategy, study design, allocation to groups, as well as censorship of animals are detailed in the Supplementary Methods (supplementary file 1).

### Prion transmission studies

Prion transmission studies were performed to assess the impact of syntaxin-6 knockout on prion disease susceptibility, propagation, and/or neurotoxicity. For susceptibility studies, mice were intracerebrally inoculated into the right parietal lobe with 30 µL of serial dilutions of RML prion-infected brain homogenate with a mixture of isoflurane and O_2_ for anaesthesia. Attack rate was the primary outcome measure, calculated as the cumulative number of incident cases of prion disease relative to the number of animals inoculated. Prion disease diagnosis was based on established clinical criteria, including sustained neurological symptoms such as ataxia, rigid tail, and impaired righting reflex, confirmed by post-mortem neuropathology. Statistical differences between genotypes were determined by binary logistic regression analysis with genotype and dose as factors. The “effective dose” was estimated using the Spearman–Karber method, described fully in the Supplementary Methods (supplementary file 1).

For studies on prion propagation and neurotoxicity, *Stx6*^+*/*+^ and *Stx6*^*−/−*^ mice were inoculated with 1% (w/v) RML prions. Survival data were analysed using Kaplan–Meier survival curves and the log-rank test. Prion infectivity titres were determined using the automated scrapie cell assay (ASCA) and SCEPA, which provide high-throughput, quantitative assessment of prion infectivity in brain homogenates. This was complemented with a cell-based assessment of neurotoxicity in primary neuronal cultures (see Supplementary Methods in supplementary file 1 for full protocols).

### Neuropathological analysis

At predefined time points or upon reaching clinical endpoints, mice were euthanized by CO_2_ asphyxiation. Brains were dissected on the sagittal plane, with one hemisphere flash frozen for biochemical analyses and the other fixed in 10% (*v*/*v*) formal buffered saline for histology. Fixed tissue was processed into paraffin blocks, sectioned at 4 µm nominal thickness, and stained for markers of prion pathology, including abnormal PrP (ICSM35), glial activation (Iba1, GFAP), and synaptic integrity (synaptophysin) using automated immunohistochemistry platforms. The Gemini AS Automated Slide Stainer was used for haematoxylin staining using a conventional approach. PrP deposition was assessed using the ICSM35 antibody, and quantification of neuroinflammation was performed using QuPath software (v0.4.3), with automated pixel classification to calculate the percentage area stained. Animals scored as non-infected by ICSM35 and H&E staining were excluded from subsequent analyses.

Detailed protocols for sample preparation, staining, and image analysis are provided in the Supplementary Methods (supplementary file 1).

### Biochemical assessment

Frozen brain homogenates (20% w/v) were prepared for proteinase K digestion or pronase digestion with subsequent western blot analysis being used to quantify total and disease-associated PrP. Samples were processed for western blotting with the ICSM35 antibody being used for PrP detection. Digital densitometry was conducted to quantify band intensities. Additional biochemical assays included assessment of neurofilament light-chain (NfL) levels in serum. Full details of the biochemical protocols, including homogenate preparation, western blot procedures, and antibodies, are provided in the Supplementary Methods (supplementary file 1).

### Transcriptomic analysis

Brain RNA was extracted and processed for next-generation sequencing as described in the Supplementary Methods (supplementary file 1), with data analysed using R and Bioconductor packages. Differential gene expression was quantified using DESeq2 and SARTools R Package. Quality control steps included RNA integrity assessment, library preparation quality control, and evaluation sequencing depth. Full procedures and bioinformatic pipelines are described in the Supplementary Methods (supplementary file 1).

### Statistical analysis

Sample sizes were determined based on power calculations for primary endpoints, with statistical analyses conducted using GraphPad Prism and InVivoStat. Where appropriate, data were transformed to meet parametric assumptions. Full statistical approaches, including criteria for exclusion and handling of missing data, are provided in the Supplementary Methods (supplementary file 1).

Full, detailed protocols for all procedures described here are available in the Supplementary Methods (supplementary file 1).

## Results

### Bidirectional manipulation of syntaxin-6 expression reveals an inverse relationship with cell-associated prion infectivity

To validate a functional role for syntaxin-6 in prion-related phenotypes in cellular models and investigate its mechanistic role, we stably knocked down *Stx6* in the prion-susceptible PK1 neuroblastoma cell line [26] by ~ 85–90% in multiple independent cell lines, relative to cell lines expressing a non-silencing control (NSC) scrambled shRNA (Supplementary Fig. 1a–b in supplementary file 2). We additionally generated two independent cell lines with ~ nine-to-tenfold overexpression of syntaxin-6 (Supplementary Fig. 1d–e in supplementary file 2). To explore whether syntaxin-6 plays a role in susceptibility to prion infection, we employed the scrapie cell assay (SCA) [26, 46], which is an ELISpot based method to quantify cell-associated prion infectivity. The output of this assay is “spot count” with the detection of foci of aggregated PrP (spots detected via the ELISpot assay) differentiating infected and non-infected cells, which allows prion titres to be calculated [26].

**Fig. 1:**
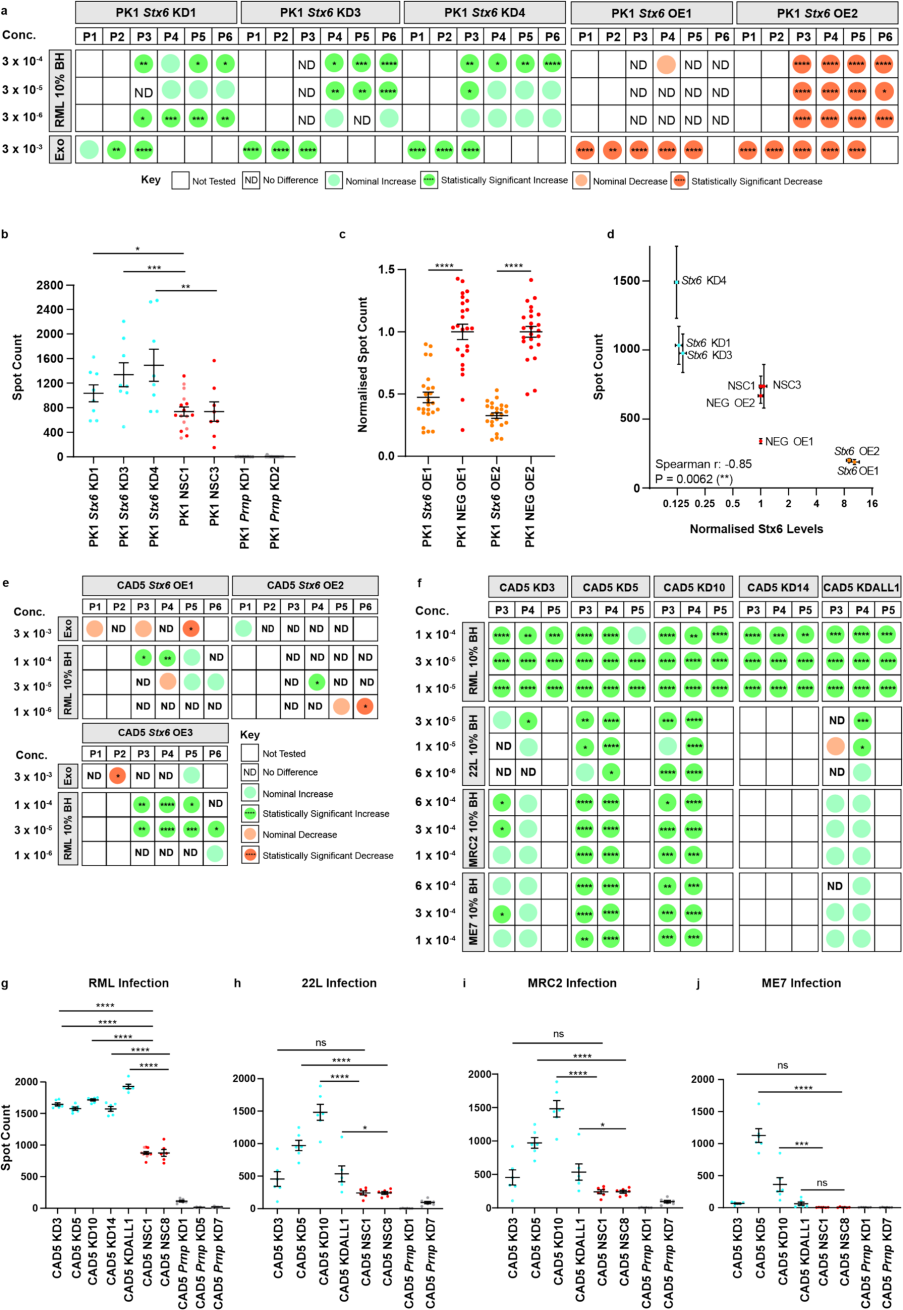
Bidirectional manipulation of syntaxin-6 expression reveals an inverse relationship with cell-associated infectivity.** a** Matrix summarising the effects of syntaxin-6 manipulation on the spot count of infected cell number in the scrapie cell assay (SCA) in PK1 neuroblastoma cells. The results of three independent PK1 *Stx6* knockdown (KD) cell lines and two PK1 independent *Stx6* overexpression (OE) cell lines are shown with each column representing a different passage number (P1–6). Each row details the infection paradigm, including different dilutions of 10% (w/v) RML-infected brain homogenate (BH) or a crude infected exosome preparation (exo). Statistics are based on one-way ANOVA followed by Fisher's LSD test of the pre-planned comparison of the relevant control (NSC or NEG OE) to each cell line with *Stx6* manipulation on a plate-by-plate basis. All statistics are based on the raw spot count except for PK1 *Stx6* overexpression cell lines, where the spot count was normalised to the haematoxylin total cell count. Nominal differences were defined as means which surpassed the threshold of the mean spot count of the negative control ± 0.5 standard deviations. **b** Representative example of the spot count of infected cell number at the 5th split in the SCA following infection with 3 × 10^–4^ RML prions (8 technical replicates/cell line). As the assay was conducted across multiple plates, spot counts of subsequent plates were normalised to the mean spot count of the non-silencing control (NSC1) on plate 1. NSC1 was technically replicated across two plates as indicated in separate colours.** c** Representative example of the spot count of infected cell number at the 3rd split in the SCA following infection with an infected exosome fraction (3 × 10^–3^; 24 technical replicates/cell line). The spot count was normalised to haematoxylin total cell count to determine the proportion of cells infected before being normalised to the mean of the relevant NEG OE cell line. **d** Graph illustrating the relationship between Stx6 protein level (normalised to the relevant negative control) and the spot count in the SCA shown in the representative examples in (**b**) and (**c**) in PK1 cells. The strength of correlation was assessed by the Spearman’s rank correlation coefficient (Spearman *r* =  − 0.85, *P* = 0.0062). **e, f** Matrix summarising the effects of syntaxin-6 manipulation on the spot count in the SCA in CAD5 catecholaminergic cells as in (**a**) with the additional interrogation of infection with other mouse-adapted prion strains, including 22L, MRC2, and ME7. **g–j** Representative examples of the spot count of infected cell number at the 4th split in the SCA following infection with 1 × 10^–5^ RML prions, 1 × 10^–5^ 22L, 6 × 10^–4^ MRC2, and 6 × 10^–4^ ME7 (6 technical replicates/cell line/strain). As the assay was conducted across multiple plates, spot counts of subsequent plates were normalised to the mean spot count of non-silencing control (NSC1) on plate 1. NSC1 was technically replicated across two plates as indicated in separate colours. Statistical differences were assessed by one-way ANOVA followed by Fisher’s LSD test on planned comparisons. *P < 0.05, **P < 0.01, ***P < 0.001, ****P < 0.0001

Infection of PK1 *Stx6* knockdown cell lines with the mouse-adapted scrapie prion strain, RML [8], resulted in a statistically robust increase in the spot count, which was broadly consistent across split numbers and prion dilutions (Fig. 1a-b). This suggests that syntaxin-6 knockdown increases the number of infected cells. In contrast, the spot count was abolished in cell lines with diminished PrP^C^ levels (PK1 *Prnp* KD1 and KD2), the substrate for conversion, confirming the expected performance of the assay (Fig. 1b). Furthermore, when we challenged PK1 *Stx6* knockdown cell lines with a limiting dose of prions, cells became susceptible to prions, in contrast to controls which were resistant to infection (Supplementary Fig. 2a–b in supplementary file 2). Strengthening these findings, infection of PK1 *Stx6* overexpression cell lines resulted in a robust reduction in the spot count (Fig. 1a, c), suggesting that overexpression may enhance clearance of prion infection. Taken together, there was a strong negative gene–dosage association between *Stx6* levels and susceptibility to prion infection across all of the aforementioned cell lines (Spearman *r* = 0.85, *P* = 0.0062) (Fig. 1d).

As we had found profoundly altered prion-related phenotypes in PK1 cells with syntaxin-6 manipulation, we additionally manipulated the expression of syntaxin-6 in a different, widely used prion-susceptible cell line, CAD5 catecholaminergic cells [40], to increase the generalisability of our findings. Therefore, 5 independent CAD5 cell lines with ~ 59%-80% knockdown of syntaxin-6 were generated (Supplementary Fig. 1g–i in supplementary file 2), as well as 3 independent cell lines with ~ 8–14-fold overexpression of syntaxin-6 (Supplementary Fig. 1k–l in supplementary file 2). Although we found no consistent effect in *Stx6*-overexpressing CAD5 cells (Fig. 1e), knockdown in CAD5 cells consistently increased the spot count after RML infection, corroborating the PK1 data (Fig. 1f–g). Strengthening these findings, this broadly extended to other mouse-adapted prion strains, including 22L (Fig. 1h), MRC2 (Fig. 1i), and ME7 (Fig. 1j) prions. Taken together, these results suggest that syntaxin-6 modifies susceptibility to prion infection in cellular models, with confidence strengthened by consistent effects across different paradigms, prion strains, and cell types.

To discount confounding factors underlying these results, we confirmed broadly comparable growth rates of the cell lines (Supplementary Fig. 1c, f, j, m in supplementary file 2) and determined that the differing spot counts were not reflective of differences in cell viability (Supplementary Fig. 2g–i in supplementary file 2). There were no consistent differences in total PrP levels (Supplementary Fig. 2j, l, m in supplementary file 2) with the exception of a subtle reduction in PK1 cells with *Stx6* overexpression (Supplementary Fig. 2k in supplementary file 2). Although this is unlikely to be a driver of the altered spot count given the modesty of the difference and the evidence that the rate of prion propagation is not a function of PrP expression in these cells [4, 14, 30, 48], we explored the underlying driver of this potential epiphenomenon. There were no differences in *Prnp* mRNA levels (Supplementary Fig. 2n in supplementary file 2) or PrP^C^ degradation kinetics (Supplementary Fig. 2o in supplementary file 2) arguing against a role for syntaxin-6 in PrP^C^ synthesis or its intracellular degradation in cellular models, respectively. Therefore, we found no evidence for confounders underlying the SCA results.

### Syntaxin-6 knockdown increases the perinuclear accumulation and alters the aggregate morphology of disease-related PrP in prion-infected cells

**Fig. 2:**
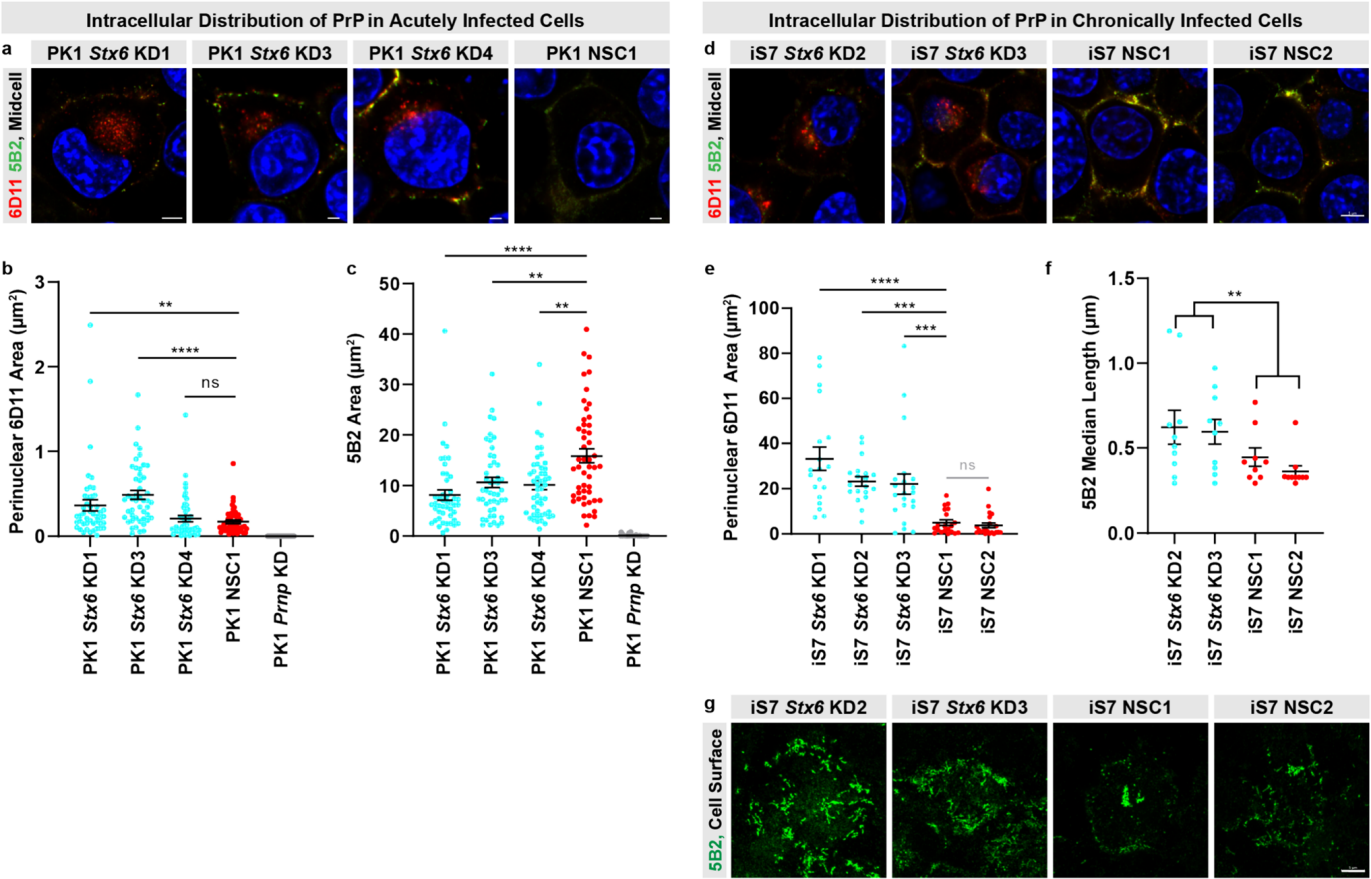
Syntaxin-6 knockdown increases the perinuclear accumulation and alters the aggregate morphology of disease-related PrP in prion-infected PK1 cells.** a** Representative images of three independent *Stx6* knockdown cell lines and one non-silencing control (NSC1) stained with the discriminatory anti-PrP antibody pair, 5B2 and 6D11, which preferentially immunolabel disease-related PrP assessed by confocal laser-scanning microscopy. DAPI, nuclear stain, blue; 6D11, red; 5B2, green. Scale bar, 5 μm (*Stx6* KD1) or 2 μm (*Stx6* KD3-4, NSC1). **b** Quantification of the 6D11 signal area in the perinuclear space normalised by total cell count as indicated by DAPI. Statistical differences were assessed by one-way ANOVA on log-transformed data followed by Fisher’s LSD test on planned comparisons. Each dot represents an individual image (*n* = 47–49/cell line). One outlier (8.28) was excluded in the graph from the *Stx6* KD1 group for visual clarity but was included in the statistical analysis of log-normal data. **c** Quantification of the total 5B2 signal area coverage at mid-cell level normalised by total cell count as indicated by DAPI. Each dot represents an individual image (*n* = 47–49/cell line). Statistical differences were assessed by one-way ANOVA on log-transformed data followed by Fisher’s LSD test on planned comparisons.** d** Representative images of iS7 cells with stable *Stx6* knockdown, or control iS7 cells expressing an NSC shRNA construct, co-stained with two anti-PrP antibodies (6D11, red and 5B2, green) as well as DAPI nuclear stain (blue) at mid-cell level. Scale bar, 5 µm.** e** Quantification of 6D11-positive area of disease-related PrP in the perinuclear space with the line representing mean ± SEM and individual dots representing an individual cell (19–21 cells/cell line). Statistical differences were assessed by one-way ANOVA followed by Fisher’s LSD test on planned comparisons. **f** Quantification of the median length of elongated disease-related PrP aggregates derived from maximum intensity projections at the plasma membrane and extracellular matrix level (9–10 images/cell line). Statistical differences were assessed on log-normal data using one-way ANOVA to test for the effect of *Stx6* expression level on length. **g** High magnification images of elongated disease-related PrP aggregates at the plasma membrane and extracellular matrix level displayed as maximal intensity projections of z-stack images to capture the length across all planes of the aggregate. Scale bar, 5 µm. For each experiment, brightness/contrast was adjusted similarly across conditions. *P < 0.05, **P < 0.01, ***P < 0.001, ****P < 0. 0001

Due to the prominent effects on the spot count, we performed complementary phenotypic characterisation of the infected *Stx6* knockdown cell lines by confocal microscopy using two antibodies, 5B2 and 6D11, which each detect a distinct disease-associated PrP staining profile in this cellular system [42]. 5B2 immunostaining recognises elongated disease-related PrP aggregates at the plasma membrane and extracellular matrix in infected cells, whereas 6D11 immunostaining is seen as punctate, perinuclear staining in addition to some plasma membrane and extracellular matrix staining. There was a statistically significant increase in the accumulation of perinuclear 6D11-positive disease-related PrP in PK1 *Stx6* KD1 and KD3 (Fig. 2a–b) as well as a statistically robust reduction in 5B2-positive plasma membrane staining in all *Stx6* knockdown cell lines (Fig. 2c). This demonstrates that syntaxin-6 knockdown redistributes disease-related PrP, consistent with a trafficking mechanism. Confocal imaging of infected *Stx6* overexpression cells revealed a universal reduction in 6D11 and 5B2 staining (Supplementary Fig. 2c–f in supplementary file 2), suggesting that syntaxin-6 overexpression was sufficient to almost clear the infection, corroborating the SCA data.

To more thoroughly explore phenotypic differences in the distribution and aggregate morphology of disease-related PrP, we additionally stably knocked down syntaxin-6 in chronically infected PK1 cells (a subclone called iS7) [42] allowing us to explore a role for syntaxin-6 in prion accumulation isolated from infection (Supplementary Fig. 3a–b in supplementary file 2). Recapitulating our observations with freshly infected cells (Fig. 2a-b), we found a prominent accumulation of perinuclear 6D11-positive disease-related PrP with *Stx6* knockdown in chronically infected cells (Fig. 2d–e). We also observed longer 5B2-positive elongated disease-related PrP aggregates at the plasma membrane and extracellular matrix with *Stx6* knockdown (*P* = 0.0062) (Fig. 2f–g) but not total load (Supplementary Fig. 3i in supplementary file 2). Taken together, these results show that syntaxin-6 knockdown causes cellular redistribution and structural reorganisation of disease-related PrP in chronically infected cells, further supporting a role for syntaxin-6 in trafficking of disease-related PrP.

### Syntaxin-6 promotes prion export in chronically infected cells

**Fig. 3:**
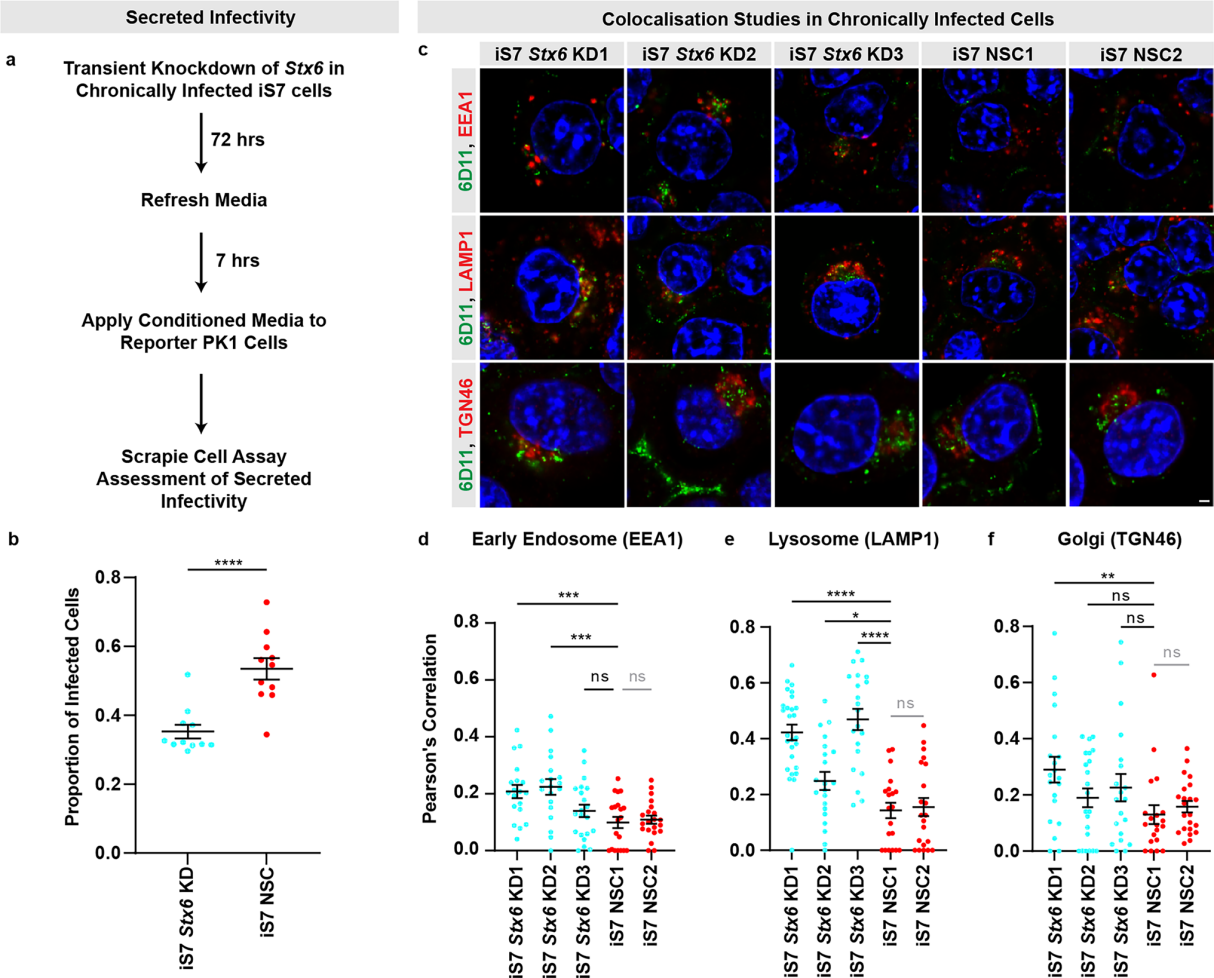
Syntaxin-6 promotes prion export in chronically infected PK1 cells. **a** Experimental design for assessing a modifying effect of syntaxin-6 knockdown on secreted infectivity from chronically infected cells (iS7 subclone). **b** The proportion of infected reporter PK1 cells (spot count normalised to haematoxylin total cell count) following the application of conditioned media harvested from iS7 cells in which *Stx6* had been transiently knocked down or a non-silencing control (NSC) shRNA had been employed. **c** Representative images of iS7 cells co-labelled with 6D11 and markers of intracellular compartments to assess for altered distribution of disease-related PrP with syntaxin-6 knockdown in the early endosome (EEA1), lysosomes (LAMP1) and in the *trans*-Golgi (TGN46). Scale bar, 2 µm. **d–f** Assessment of levels of colocalisation between 6D11-positive PrP and organelle markers, expressed as Pearson’s correlation coefficient for EEA1 (*n* = 19–21 cells/cell line), LAMP1 (*n* = 20–27 cells/cell line) and TGN46 (*n* = 20–22 cells/cell line). Line represents mean ± SEM with one-way ANOVA followed by Fisher’s LSD test being used to test statistical differences. For each experiment, brightness/contrast was adjusted similarly across conditions. *P < 0.05, **P < 0.01, ***P < 0.001, ****P < 0. 0001

To test whether a role for syntaxin-6 in prion export could explain the altered spot counts, we transiently knocked down syntaxin-6 in chronically infected iS7 PK1 cells followed by collecting conditioned media to infect PK1 reporter cells (Fig. 3a). We observed a reduction in secreted infectivity after syntaxin-6 knockdown, measured by the SCA (18.2% ± 3.7% reduction, mean ± SEM) (Fig. 3b), suggesting that syntaxin-6 mediates prion export. Corroborating this, when we harvested media from the infected stable *Stx6* knockdown and overexpression PK1 cell lines described previously, syntaxin-6 knockdown resulted in reduced relative secreted infectivity titres, with the converse being observed with syntaxin-6 overexpression, following correction for baseline differences in cell-associated infectivity (Supplementary Fig. 2p, q in supplementary file 2).

In further support for a role of syntaxin-6 in prion export, as opposed to involvement in an intracellular trafficking step, we observed a generalised increase in the degree of colocalisation of 6D11-positive disease-related PrP with markers of intracellular organelles (Fig. 3c–f). Syntaxin-6 knockdown resulted in increased colocalisation of 6D11 with the early endosome marker, EEA1, (Fig. 3d) and the lysosome marker, LAMP1, (Fig. 3e) with some additional evidence for the TGN marker, TGN46 (Fig. 3f).

Collectively, this provides evidence for prion export being the syntaxin-6-driven molecular susceptibility mechanism.

### Syntaxin-6 modifies prion pathogenesis in vivo by modulating the risk of disease development

As we had established a role for syntaxin-6 in modulating prion-related phenotypes in cellular models, we next wanted to validate a functional role for syntaxin-6 in prion pathogenesis in vivo and explore the disease stage at which it acts. The “gold standard” paradigm for studying prion disease pathogenesis is prion transmission in mice. Mice are naturally susceptible to prion infection, developing *bona fide* disease with faithful recapitulation of the clinical and neuropathological hallmarks of human disease when experimentally inoculated with prions such as the mouse-adapted scrapie prion strain, RML [8]. To determine whether *Stx6* knockout modified the risk of mice developing prion disease, we intracerebrally infected *Stx6*^+*/*+^ and *Stx6*^*−/−*^ mice (*n* = 90/genotype) with a tenfold serial dilution series of 10% (w/v) RML prion-infected brain homogenate and assessed disease development using both clinical and neuropathological diagnoses. The focus of this study was to examine prion doses with a partial attack rate (defined as < 90% across both arms of the study: 10^–5^, 10^–6^, 10^–7^, 10^–8^), where the likelihood of disease development was uncertain, providing a paradigm to assess whether syntaxin-6 modulated that risk.

**Fig. 4:**
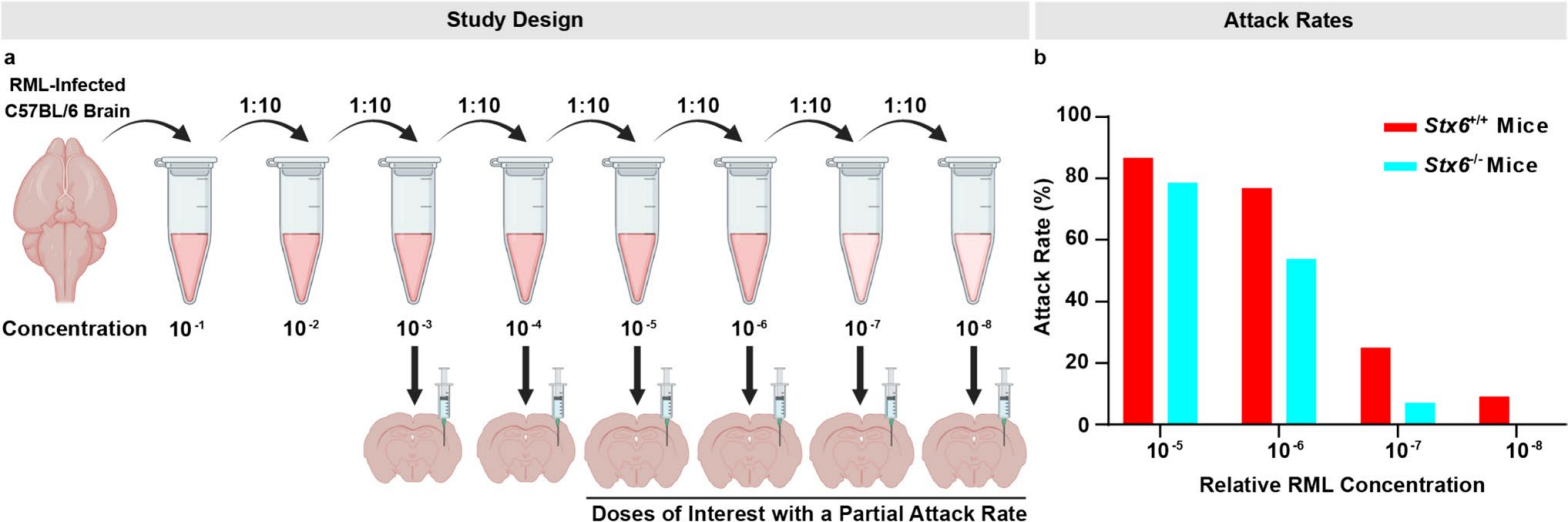
Syntaxin-6 knockout renders mice less susceptible to prion disease when administered low doses of prions.** a** Experimental design of intracerebral inoculation of *Stx6*^+/+^ and *Stx6*^*−/−*^ mice with a tenfold dilution series of 10% (w/v) RML prion-infected brain homogenate (*n* = 15/genotype/dose) with prion doses with a partial attack rate (< 90%) being of interest. Created in BioRender. One, S. (2025). https://BioRender.com/t36g169. **b** Bar chart showing the attack rates of prion disease in mice administered 10^–5^, 10^–6^, 10^–7^, or 10^–8^ concentrations of RML prions

For each of the 10^–5^, 10^–6^, 10^–7^, and 10^–8^ concentrations, the proportion of prion disease cases relative to the animals surviving to the end of the study (attack rate, see methods) was consistently lower in *Stx6*^*−*/−^ mice relative to *Stx6*^+*/*+^ mice, suggesting that they were more resistant to disease development (Table 1; Fig. 4). Logistic regression analysis demonstrated a significant effect of dose (*P* < 0.0001) and genotype (*P* = 0.05) on prion disease diagnosis, with infected *Stx6*^+*/*+^ mice having 2.19 [95% CI 1.01–4.56] times higher odds of developing prion disease compared to *Stx6*^*−/−*^ animals at concentrations 10^–5^ and lower. This suggests syntaxin-6 knockout reduces susceptibility to prion infection, which is further supported by calculations estimating the “effective” dose administered to *Stx6*^+*/*+^ and *Stx6*^*−/−*^ mice, which was reduced in *Stx6*^*−/−*^ mice by a log order of magnitude relative to *Stx6*^+*/*+^ mice (10^5.82^ LD_50_/mL and 10^6.61^ LD_50_/mL, respectively) by the Spearman–Karber method or ~ 0.5 log difference with the Reed and Muench method.

**Table 1:** Assessment of susceptibility differences in *Stx6*^+*/*+^ and *Stx6*^*−/−*^ mice inoculated with RML prion dilutions with a partial attack rate

Concentration	*Stx6*^+/+^ mice		*Stx6*^*−/−*^ mice		Odds Ratio (95% CI)	*P* value (genotype)
	Attack rate	Attack rate (%)	Attack rate	Attack rate (%)		
10^–5^	13/15	86.7	11/14	78.6	1.77 (0.309–11.2)	0.0500
10^–6^	10/13	76.9	7/13	53.8	2.86 (0.509–12.7)	
10^–7^	3/12	25.0	1/14	7.14	4.33 (0.537–59.9)	
10^–8^	1/11	9.09	0/15	0.00	Infinity (0.152-infinity)	
Combined	27/51	52.9	19/56	33.9	2.19 (1.01–4.56)	

Attack rates of *Stx6*^+*/*+^ and *Stx6*^*−/−*^ mice infected with dilutions of 10% (w/v) RML prion-infected brain homogenate with a partial attack rate (defined as < 90% across both arms of the study). Attack rate was defined as the total of affected mice as a proportion of the number of inoculated mice, excluding mice which died due to unrelated health concerns or animals where there was a discrepancy between the clinical and pathological observations. Calculated odds ratios are shown with 95% confidence intervals (CI) determined by the Baptista–Pike method [2]. The *p* value refers to the results of logistic regression analysis with prion dose and genotype as factors

Therefore, in addition to a role for syntaxin-6 in altering prion-related phenotypes in cellular models, this established a role for syntaxin-6 in modulating early stages of prion pathogenesis in vivo.

### Syntaxin-6 does not alter prion propagation or prion-induced neurotoxicity in vivo during established disease

**Fig. 5:**
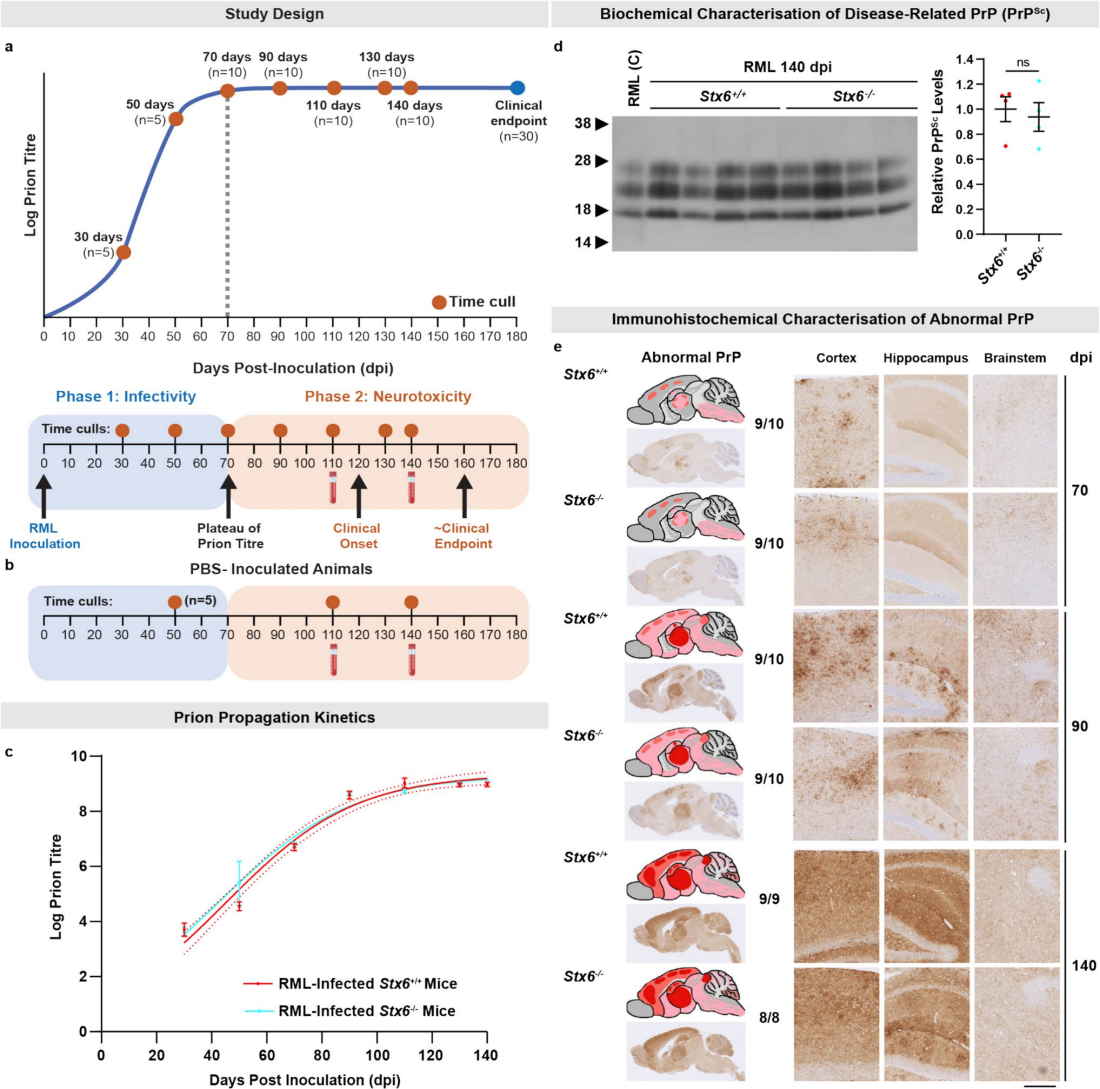
Syntaxin-6 knockout has no effect on prion propagation kinetics or levels of disease-related PrP in mice infected with RML prions.** a** Experimental design mapped onto the two-phase kinetics model whereby RML-infected *Stx6*^+*/*+^ and *Stx6*^*−/−*^ mice were culled at predefined time points to assess for differences in prion propagation and neurotoxicity-related outcome measures. **b** Timed culls of PBS-inoculated controls. **c** Prion titres [log tissue-culture infectious units (TCIU) per gram brain] of RML-infected *Stx6*^+/+^ and *Stx6*^*−/−*^ mice (n = 5–10/genotype/time point), across the incubation period (dpi, days post-inoculation). All PBS-inoculated brain homogenates were negative for infectivity (not shown). Curves were fitted using the logistic growth model [goodness of fit, *r*^2^: 0.928 (*Stx6*^+*/*+^), 0.926 (*Stx6*^*−/−*^)]. Bars indicate mean ± SEM with dotted lines representing 95% confidence intervals. **d** 10% (w/v) brain homogenates from RML-infected *Stx6*^+*/*+^ and *Stx6*^*−/−*^ mice at 140 dpi were analysed by immunoblotting with the anti-PrP antibody, ICSM35, after digestion with proteinase K (50 µg/mL, 37 °C, 1 h). Semi-quantification of the PrP^Sc^ signal was performed using densitometry with each sample being normalised to the average of RML-infected *Stx6*^+*/*+^ mice. Bar graphs represent mean ± SEM of 4 biological replicates/genotype. C, positive control RML sample. Statistical differences were tested with a Student’s *t* test (*P* = 0.693). The image brightness/contrast was optimally adjusted. **e** Spatiotemporal differences of PrP deposition were assessed by immunohistochemistry using the anti-PrP antibody, ICSM35, at 70, 90, and 140 days post-inoculation (dpi) (*n* = 9–10/genotype). A schematic is shown to represent the overall staining pattern in the respective groups (pale pink: mild PrP deposition; pink shading: moderate PrP deposition; red shading: intense PrP deposition). Representative images of the whole brain section as well as magnified images from the cortex, hippocampus, and brainstem are shown. Scale bar, 2.5 mm (overview) and 0.5 mm (zoom). Numbers shown next to the schematics report the number of animals in the group positive for the pathology shown as a fraction of the total number of animals in each group

This modifying effect of syntaxin-6 on disease susceptibility in vivo could either be acting through directly modulating the establishment of prion infection, or alternatively, by altering subsequent prion propagation or prion-induced neurotoxicity. To systematically interrogate a role for syntaxin-6 in prion replication and neurotoxicity*,* we infected *Stx6*^+*/*+^ and *Stx6*^*−/−*^ mice (*n* = 110/genotype) with 1% (w/v) RML prion-infected brain homogenate, with animals subsequently being culled at multiple predefined time points or at the onset of clinical disease (Fig. 5a). This allowed age-matched, cross-sectional analyses of prion-related phenotypes in the evolving stages of disease, with timed culls of PBS-inoculated mice providing the negative control (Fig. 5b).

The automated SCA (ASCA) [26, 46] was used to provide measurement of prion titres throughout the disease course (Fig. 5c). Prion titres in both RML-infected *Stx6*^+*/*+^ and *Stx6*^*−/−*^ mice increased rapidly at comparable rates before reaching a similar maximal prion titre of ~ 10^8.5^ infectious units/g at ~ 90 days post-inoculation (dpi), in line with the two-phase kinetics model [43, 44]. This suggests that syntaxin-6 does not alter prion propagation kinetics in established disease. This was further supported by biochemical assessment of disease-related proteinase K (PK)-resistant PrP (PrP^Sc^) at 140 dpi, which was comparable in infected *Stx6*^+*/*+^ and *Stx6*^*−/−*^ mice, with indistinguishable electrophoretic mobility and glycosylation patterns (Fig. 5d). This was also corroborated by immunohistochemical detection of disease-related PrP, where we found no differences in the appearance, extent and distribution of PrP deposits with both the onset and evolution of deposition being broadly comparable in RML-infected *Stx6*^+*/*+^ and *Stx6*^*−/−*^ mice (Fig. 5e). Taken together, these results suggest that syntaxin-6 is not involved in prion propagation nor in modulating the levels of disease-related PrP during established disease.

Subsequently, we explored whether there were any differences in the onset and/or progression of markers of neurotoxicity or neurodegeneration in RML-infected *Stx6*^+*/*+^ and *Stx6*^*−/−*^ mice. There were no differences in the extent or distribution of intraneuronal vacuoles (“spongiosis”) (Supplementary Fig. 4a–b in supplementary file 2), synaptic integrity (Supplementary Fig. 4c in supplementary file 2), or the spatiotemporal evolution of astrogliosis and microgliosis (Supplementary Fig. 4d–e in supplementary file 2) across the disease course. Furthermore, we observed comparable levels of disease-associated PK-sensitive PrP species (Supplementary Fig. 5a–b in supplementary file 2) and toxicity levels in prion-infected brain homogenates using a validated neurotoxicity assay [3] (Supplementary Fig. 5c–d in supplementary file 2). There were also no consistent differences in serum neurofilament light-chain (NfL) levels (Supplementary Fig. 5e in supplementary file 2), which is a sentinel neurodegeneration biomarker in prion disease [33]. Finally, RML-infected *Stx6*^+*/*+^ and *Stx6*^*−/−*^ mice exhibited comparable neurological phenotypes, time to first symptom (*Stx6*^*−/−*^ median [95% confidence interval] = 129 days [126–129] vs. *Stx6*^+*/*+^  = 129 days [126–131]), incubation times (*Stx6*^*−/−*^ median [95% confidence interval] = 139 days [134–141] vs. *Stx6*^+*/*+^  = 140.5 days [139–144]), and clinical progression (Supplementary Fig. 5f–h in supplementary file 2). These results suggest that syntaxin-6 is not involved in prion-induced neurotoxicity.

Brain transcriptomic analyses of *Stx6*^+*/*+^ and *Stx6*^*−/−*^ mice suggested that compensatory mechanisms were not at play at the RNA level with there being no significant upregulation of genes encoding other syntaxins/trafficking proteins (Supplementary Table 1 in supplementary file 3). Therefore, taken together with the positive results of the titration study, these findings suggest syntaxin-6 modifies the establishment of disease, with no discernible effect on prion propagation nor prion-induced toxicity in established disease.

## Discussion

**Fig. 6:**
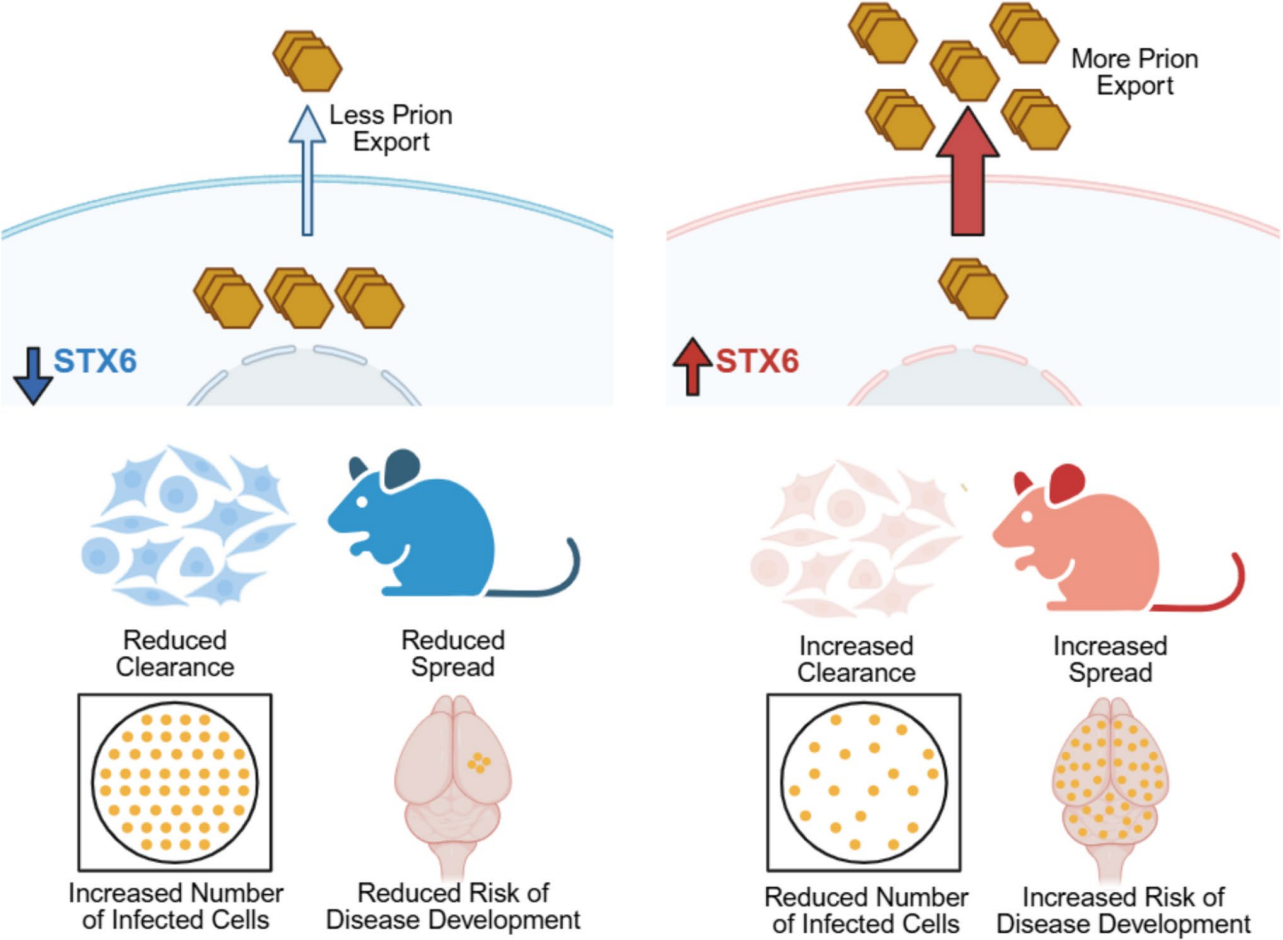
Speculative model proposing a role for syntaxin-6 in prion disease pathogenesis. Cellular and in vivo evidence points to a role for syntaxin-6 in prion trafficking and export. In cells, this paradoxically provides a clearance mechanism. In contrast, we propose that this enhances spread in vivo, increasing the risk of disease development. Figure created using BioRender.com

We show that syntaxin-6 modifies prion pathogenesis in cell and mouse models, providing functional support for previous GWAS findings and mechanistic insight into the risk conferred by syntaxin-6 variants in sCJD. We show that syntaxin-6 knockout in vivo reduces susceptibility to prion infection, suggesting a role for syntaxin-6 in disease establishment. We provide evidence for altered intracellular trafficking and export of prions as a cellular susceptibility mechanism which may underlie this observation (Fig. 6).

In cellular models, syntaxin-6 levels inversely correlated with prion infection levels with syntaxin-6 knockdown increasing cell-associated infectivity. This was recapitulated with *Stx6* manipulation in several cell lines infected with a multitude of different prion strains, demonstrating the generalisability of these findings. Underlying this, syntaxin-6 knockdown diminished prion infectivity in conditioned media, consistent with a proposed function of syntaxin-6 in the export of proteins from cells [21, 38], a key mechanism of prion spread [17]. This points to a cellular susceptibility mechanism linked to prion export, which enhances prion clearance from infected cells. This is further supported by our results in cells stably overexpressing syntaxin-6 which show a remarkable diminishment of cell-associated prion levels.

Syntaxin-6 knockdown in cells additionally resulted in a conspicuous redistribution of disease-associated PrP to a perinuclear compartment, in keeping with syntaxin-6 being involved in trafficking of disease-associated PrP. Indeed, previous work showing disease-associated PrP colocalises with syntaxin-6 provides a biologically plausible basis for this [45]. However, we did not find evidence of a block at any specific intracellular trafficking step; instead, we observed evidence of increased disease-associated PrP colocalisation with markers of every intracellular compartment examined. This general increase in intracellular disease-associated PrP provides additional evidence that syntaxin-6 mediates prion export.

We additionally observed longer disease-associated PrP aggregates at the plasma membrane in chronically infected cells with syntaxin-6 knockdown, suggesting enhanced fibrillisation kinetics. This aligns with previous *in vitro* work, suggesting that syntaxin-6 inhibits the ordered formation of PrP fibrils through direct binding [45]. Despite observing profound phenotypic differences in chronically infected cells with syntaxin-6 knockdown, there were no changes in total prion load during established infection, in line with the in vivo results where we only found effects of syntaxin-6 manipulation on disease risk. Collectively, the cell-based work supports a role for syntaxin-6 in disease-associated PrP trafficking and export, further supporting the contention that vesicle trafficking is a key mechanism contributing to prion pathogenesis [1, 11, 18, 36, 47].

Our in vivo data further validate a role for syntaxin-6 in prion pathogenesis, acting as a modifier only in early disease stages. The influence of syntaxin-6 on prion susceptibility in prion-infected mice aligns with the human genetic data linking increased expression of syntaxin-6 to higher disease risk [24, 27]. In contrast, knockout did not affect prion propagation kinetics or neurotoxicity in established disease, mirroring the human findings that *STX6* risk variants do not impact rates of sCJD progression [20]. This aligns with other work suggesting a distinction between genetic susceptibility factors and drivers of disease progression [28, 29, 34]. Given that our cellular studies implicate syntaxin-6 in prion trafficking and export, we propose that reduced prion secretion resulting from syntaxin-6 knockout limits extracellular prion availability and thereby restricts intercellular spread in vivo, lowering the risk of disease establishment. Paradoxically, in cell culture the reduction in prion export increases intracellular accumulation and infectivity. This illustrates how the same underlying mechanism (altered prion trafficking) can lead to divergent outcomes depending on the biological context (Fig. 6).

Therapeutic targets supported by human genetic evidence are more likely to translate successfully [32, 35, 37, 39]. Although PrP lowering offers a genetically validated therapeutic approach in prion disease, with preclinical efficacy [41] and target engagement/safety demonstrated in humans [31], extending our therapeutic repertoire would be valuable. Our results suggest that the modifying effects of syntaxin-6 do not lend themselves well to drug development in symptomatic patients. Indeed, we show that syntaxin-6 reduction had no effect in established mouse prion disease. Given its early modifying effect, *STX6*-lowering could potentially be useful in prevention in the at-risk patient population, such as genetic mutation carriers. This resonates with recent work, suggesting that targeting GWAS-identified susceptibility factors is more likely to yield efficacy in pre-symptomatic populations [34].

Limitations of this work include the use of acquired prion infection models, which differ from the spontaneous disease initiation seen in sporadic human cases. Nonetheless, given the lack of more representative models, these studies provide a valuable proof-of-concept for functional validation of genetic risk mechanisms. The effect sizes observed were modest, but this is consistent with the underlying human genetic data, where the associated risk variants are statistically robust, but confer only a ~ 15% increase in disease susceptibility. Together with the genetic evidence, our findings strongly support a contributory role for syntaxin-6 in prion pathogenesis.

In conclusion, we identify syntaxin-6 as a modifier of early prion disease pathogenesis in vivo and demonstrate its role in regulating the trafficking and export of prions. This work strengthens the case for syntaxin-6 as a pleiotropic risk factor in neurodegenerative disease, anchored in human genetics, and sheds light on the specific stage of disease at which it acts. Moreover, it highlights prion export as a fundamental susceptibility mechanism in prion biology and, potentially, in broader protein misfolding disorders.

## Supplementary Information

Below is the link to the electronic supplementary material.

Supplementary file1: Supplementary methods (DOCX 83 KB)

Supplementary file2: Supplementary data (DOCX 9960 KB)

Supplementary file3: Supplementary table (XLSX 14 KB)
