# Correction: Intracellular trafficking SNARE protein, syntaxin-6, modifies prion cellular phenotypes and risk of disease development in vivo

**DOI:** 10.1007/s00401-026-03003-8

**Published:** 2026-03-30

**Authors:** Elizabeth Hill, Mitali M. Patel, Juan M. Ribes, Jacqueline Linehan, Fuquan Zhang, Tatiana Jakubcova, Shyma Hamdan, Andrew Tomlinson, Tiziana Ercolani, Christian Schmidt, Parvin Ahmed, George Thirlway, Fabio Argentina, Aline T. Marinho, Emma Jones, Nicholas Kaye, Craig Fitzhugh, Graham S. Jackson, Sebastian Brandner, Peter-Christian Klöhn, John Collinge, Thomas J. Cunningham, Simon Mead

**Affiliations:** 1 MRC Prion Unit at UCL, UCL Institute of Prion Diseases, Courtauld Building, 33 Cleveland Street, London, W1W 7FF UK; 2 School of Biochemistry, Biomedical Sciences Building, University of Bristol, Bristol, BS8 1TD UK; 3 Department of Neurodegenerative Disease, UCL Queen Square Institute of Neurology, London, WC1N 3BG UK

**Correction: Acta Neuropathologica (2025) 150:48** 10.1007/s00401-025-02946-8

In the original version of this article, the given and family name of the authors was incorrectly written as “Mitali Patel” and “Peter Kloehn” but the correct name should be “Mitali M. Patel” and “Peter-Christian Klöhn”.

The original article has been corrected.

