# Supplementary material for: Intracellular trafficking SNARE protein, syntaxin-6, modifies prion cellular phenotypes and risk of disease development in vivo
Source: Acta Neuropathol. 2025 Nov 4;150(1):48. doi: 10.1007/s00401-025-02946-8 (PMC12586215; doi:10.1007/s00401-025-02946-8)
Supplement: Supplementary file 1 — Supplementary file1: Supplementary methods (DOCX 83 KB) [file 401_2025_2946_MOESM1_ESM.docx]

# Supplementary Methods

**Statistical Approach**

The broad statistical approach for each study is outlined but the specific ‘n’ number and any deviations in the statistical analyses are detailed for each specific experiment in the relevant figure legend. All graphing and statistical analysis were conducted in Graphpad Prism or InVivoStat.

**Research Governance**

All experimental procedures employing mouse-adapted prions were conducted in microbiological containment level 2 (CL2) or level 3 (CL3) facilities with strict adherence to safety protocols and guidelines. Work with mice was performed under approval and license granted by the UK Home Office (Animals (Scientific Procedures) Act 1986), which conforms to UCL institutional guidelines and Animal Research: Reporting of In Vivo Experiments (ARRIVE) guidelines (www.nc3rs.org.uk/ARRIVE/).

**General Animal Maintenance and Husbandry**

**Housing Conditions and Animal Care**

Breeding colonies were kept in individually ventilated cages at a temperature of 20-24°C, humidity of 45-65%, an average of 75 air exchanges per hour in the cages and a 12/12-hour light/dark cycle with the lights on at 7AM. The maximum caging density was five mice starting from weaning. Lignocel or EcoPure wood fibres were provided as bedding along with ‘Bed r’ nest’ nesting materials, wood blocks and a shelter.

Mice were fed a standardised irradiated mouse diet and provided with reverse osmosis drinking water *ad libitum.* All materials, including cages, lids, feeders and water bottles were washed in a cage washer and autoclaved before use. All mice were frequently checked by veterinary and animal care staff and had not undergone prior procedures. All animals otherwise received no procedures except those reported in this work. Work was conducted in a CL3 facility, with staff required to air shower upon entry and exit. External animals were only brought in from other clean-screened units and following in-house vet approval.

**Genotyping**

DNA was extracted from ear biopsies of mice using MyTaq Extract-PCR Kit with a 30 min incubation at 75°C followed by a 15 min incubation at 95°C. PCR amplification was performed using 1x MyTaq HS Red Mix with a triple primer set. This was amplified using a Biorad Tetrad 2 (95°C for 3 min, 35x(95°C for 15 sec, 59°C for 15 sec, 72°C for 20 sec), 72°C for 5 min). PCR reactions were analysed by agarose gel electrophoresis (0.5% agarose in 1x TAE with gelred run at 4.6 V/cm) with image capture performed on a Universal Hood II Gel Doc System.

**Prion Transmission Studies into Mice**

**Breeding Strategy**

The *Stx6^+/+^* and *Stx6^-/-^* C57BL/6N homozygous lines [10] were first intercrossed to mitigate the effects of genetic drift, with heterozygote mice subsequently being intercrossed to generate littermate controls to populate the clinical endpoint groups of the 1% (w/v) RML transmission study. Clinical endpoint animals were randomly allocated to groups based on Mendelian inheritance. For reasons of feasibility, re-established homozygous independent lines were used to populate the other RML timed cull groups as well as the RML titration study. Animals were randomised by cage when assigning to groups.

**Study Design, Power Calculations and Statistical Analyses of Prion Transmission Studies**

*Serial Dilution of RML into Stx6^+/+^ and Stx6^-/-^ Mice*

Mice were infected with a dilution series of 10% (w/v) RML-infected brain homogenate with the attack rate being the primary outcome measure for concentrations with a <90% partial attack rate (10^-5^, 10^-6^, 10^-7^, 10^-8^). Diagnosis was based on the clinical and neuropathological observations (PrP immunostaining as the main assessment with H&E assessment of spongiosis of some PrP-negative animals to validate it was not a technical error). Animals which were culled due to unrelated health concerns before the elective 400 days post-inoculation (dpi) time cull were excluded, as well as animals where there was a discrepancy between the clinical and neuropathological observations (9 *Stx6^+/+^* animals and 4 *Stx6^-/-^* animals). This was a female-only study to allow comparability with previous experiments. The main comparison in these studies were prion-infected *Stx6*^+/+^ mice with *Stx6^-^*^/-^ mice at each dose administered. A single animal was considered an experimental unit.

As this was the first study of this kind in *Stx6^-/-^* mice, we proposed a shift in the attack rate by a 1 log dilution as a hypothesis on which to base power calculations. For example, wild type C57BL/6 mice infected with RML prions have an attack rate of 100% when inoculated with 10^-6^ RML concentration and a 20% attack rate with 10^-7^ concentration[18]. Based on our hypothesis, these numbers were used to determine the sample size using Fisher's exact test (two sided; 90% power). The suggested 8 animals/group, which was increased to 15 in line with precedent in similar types of studies and to allow for losses due to incurrent illness. This provided 90% power (2-sided) to detect a change in attack rate from 100% to 56%, or from 80% to 29% at a single dilution level.

*Two-Phase Kinetics RML Transmission Study in Stx6^+/+^ and Stx6^-/-^ Mice*

Following inoculation with 1% (w/v) RML prions, female *Stx6*^+/+^ and *Stx6*^-/-^ mice were culled at multiple predefined time points or at onset of clinical disease**.** As controls, some mice were inoculated with Dulbecco’s Phosphate Buffered Saline (DPBS, –Mg^2+^ and Ca^2+^). This was a female-only study to allow comparability with previous experiments. The main comparison in these studies were prion-infected *Stx6*^+/+^ mice with *Stx6^-^*^/-^ mice at each time point. A single animal was considered an experimental unit.

Group sizes for clinical endpoint were determined through power calculations using the effect size and variability from published work[10] (two-sided; 80% power). The suggested 22 animals/group was increased to 30 to allow for losses due to unrelated health concerns. Group sizes for the scheduled time culls were based on estimated effect sizes of the primary outcome measures, with 10 animals/arm giving 90% power to detect an 15% change in prion titre and with 5 animals/arm giving 90% power to detect a 21% change in prion titre.

Unless stated otherwise in the figure legend, statistical differences of the biochemical data were assessed by an unpaired t-test between infected *Stx6*^+/+^ and *Stx6*^-/-^ mice as the main experimental comparison. For neuropathological studies assessing multiple brain regions for each animal, a 2-way repeated measures mixed model approach was used for statistical analysis using the unstructured covariance structure to model the within-subject correlations, with genotype as the treatment factor and brain region as the repeated factor.

In some of the exploratory studies (neurotoxicity assay, biomarker assessment), one-way ANOVA was performed followed by Fisher’s LSD test of the following pre-planned comparisons:

1. Differences between infected *Stx6*^+/+^ and *Stx6*^-/-^ mice as the main experimental comparison.
2. Differences between RML-infected *Stx6*^+/+^ mice relative to PBS-inoculated *Stx6*^+/+^ controls to establish a disease-associated deficit.
3. Differences between PBS-inoculated *Stx6*^+/+^ mice and PBS-inoculated *Stx6*^-/-^ mice to assess for baseline differences.

To satisfy the parametric test assumptions, transformations were conducted in some cases, as detailed in the figure legends.

One *Stx6^+/+^* and one *Stx6^-/-^* animal in the 50 dpi experimental group died following the procedure of inoculation so had to be removed from the study. Animals diagnosed with prion disease prior to their scheduled time cull were excluded from follow-up analysis as they were not age-matched (1 *Stx6*^+/+^ animal at 130 dpi and 1/genotype at 140 dpi).

**Inoculation and Neurological Observation**

10% (w/v) RML-infected C56BL/6 brain homogenate was diluted to 1% (w/v) in DPBS. Any subsequent dilutions for lower infecting doses were performed using 1% (w/v) normal C57BL/6 brain homogenate in DPBS as diluent. Brain homogenate dilutions were prepared on the same day and frozen at -80°C until infection. 6-9-week-old mice were anaesthetized with a mixture of isoflurane and O_2_ and intracerebrally inoculated into the right parietal lobe with 30 µL of prion-infected brain homogenate or DPBS, as previously described[15, 22]. Matched groups (set time cull or set prion dose) were infected in the same inoculation session to reduce confounding factors or split into two inoculation batches with a balanced genotype ratio if the former was unfeasible.

Following inoculation, all mice were examined daily for early indicators of clinical prion disease including piloerection, sustained erect ears, intermittent generalized tremor, unsustained hunched posture, rigid tail, mild loss of coordination, and clasping hind legs when lifted by the tail. Definite diagnosis of clinical prion disease was made when mice exhibited any 2 early indicator signs in addition to 1 confirmatory sign, or any 2 confirmatory signs, marking the experimental endpoint. The confirmatory signs included ataxia, impairment of righting reflex, dragging of hind limbs, sustained hunched posture, or significant abnormal breathing. Technical staff who made judgements on diagnosing prion disease were blinded to study design and endpoints were called by the same observing team.

**Humane and Elective Time Culls**

All animals were culled upon definite prion disease diagnosis or at a defined, elective timed cull. Any animals that did not develop clinical prion disease were culled at 400 dpi with post-mortem assessment of prion-related neuropathological features. Animals were not followed up beyond this to avoid misdiagnosis due to the overlap of prion disease symptoms and general, non-specific characteristics of aged mice. Animals were sacrificed by CO_2_ asphyxiation. Brains were removed, dissected on the sagittal plane with the right hemisphere flash frozen and stored at -80^o^C, whilst the left hemisphere was fixed in 10% (v/v) formal buffered saline. Terminal blood was taken via post-mortem cardiac puncture and serum prepared by centrifuging for 10 min at 2600 rpm after allowing 10 min for clotting in Sarstedt Microvette Serum tubes.

**Endpoint Survival Statistical Analyses**

*Attack Rate Analysis in the Serial Dilution of RML Transmission Study*

Attack rate was calculated as the cumulative number of incident cases of prion disease relative to the number of animals inoculated. Statistical differences between genotypes were determined by binary logistic regression analysis with genotype and dose as factors. This was complemented by calculating differences in the odds ratio of prion disease development across the different groups using the Baptista-Pike method to determine confidence intervals.

*Estimation of the “Effective Dose” Using the* *Spearman-Karber Method in the Serial Dilution of RML Transmission Study*

To estimate the “effective” dose infected into *Stx6^+/+^* and *Stx6^-/-^* mice in the RML serial dilution study, an adaption of the Spearman-Karber method[7] using the following equation and definitions:

Log_10_ 50% end point dilution = - (x_o_ – d/2 + d ∑ r_i_/n_i_).

X_0_ = log_10_ of the reciprocal of the highest dilution where there was an attack rate of >90%. D = log_10_ of the dilution factor. N_i_ = average number of animals in each individual dilution after discounting unrelated deaths. ∑R_i_, = sum of total number of positive animals (out of n_i_) with summation started at x_0_.

*Comparison of Time to First Symptom and Time to Definite Clinical Prion Disease Diagnosis*

In the two-phase kinetics RML prion transmission study, disease-free incubation period was defined as the number of days from inoculation to the onset of the first prion disease-associated neurological symptom. Incubation period was defined as the number of days from inoculation until definite prion disease diagnosis[15]. Log-rank test survival analysis and the Kaplan-Meier method were employed to test for differences in the median time of these two parameters. Due to the anticonservative nature of the statistics, a 20% difference was defined as a biologically meaningful effect size. Animals culled due to unrelated health problems or that remained alive at the end of the study without a prion disease diagnosis were censored.

Differences in disease duration were estimated as the difference between the time of first symptoms and definite prion disease diagnosis.

**Immunohistochemical Assessment**

The fixed left hemisphere of the brain was embedded in paraffin wax followed by serial sectioning (4 μm nominal thickness) as previously described[9, 20]. Sections were then deparaffinised prior to investigation of abnormal PrP (ICSM35 antibody, 1:1000), spongiosis (H&E), microgliosis (Iba1 antibody; 1:1000 or 1:250), astrocytosis (GFAP antibody: 1:1000) and synapse loss (anti-synaptophysin antibody; 1:5000) on the Ventana Discovery XT automated IHC staining machine with haematoxylin as the counterstain. Sections were treated using Ventana proprietary detection reagents before staining utilizing 3,3′-diaminobenzidine tetrahydrochloride as the chromogen (DAB Map Detection Kit). The Gemini AS Automated Slide Stainer was used for haematoxylin staining using a conventional approach. Slides were digitally scanned on a LEICA SCN400F scanner and the NanoZoomer 360 at ×40 magnification, images captured from the NDP.serve3 or NZConnect software and composed with Adobe Photoshop.

Animals were scored as positive or negative for abnormal PrP. For H&E, neuronal loss was scored in the hippocampus using 4-point score. Whole brain region immunostaining of GFAP and Iba1 was quantified using QuPath (v0.4.3) software with the experimenter blinded to the genotype of the sections. Colour deconvolution was performed followed by pixel classification to select tissue for analysis (whole brain). Artifacts were manually removed before pixel classification being applied to select regions positive for DAB staining allowing calculation of the % area stained relative to total tissue area analysed. A similar analysis was also done specifically in the thalamus, cortex and hippocampus, brain regions implicated in prion disease, with analysis being performed following manual annotation of the brain regions. Animals scored as non-infected by ICSM35 and H&E staining were excluded from the analysis.

**Biochemical Assessment**

20% (w/v) homogenates were prepared in DPBS by ribolysing with 1.4 mm ceramic homogenisation beads at 6,500 rpm for 45 sec using the Ribolyser Precellys 24. Homogenates were stored at -80°C until use.

For immunoblotting, 1 μL benzonase was incubated with 20 μL aliquots of 10% (w/v) brain homogenates in DPBS. Following a 1 hr incubation with proteinase K (PK, 50 μg/mL) at 37°C and 800 rpm, samples were mixed with 2X sample buffer (125 mM Tris-HCl, 20% (v/v) glycerol, pH 6.8, containing 4% (w/v) SDS, 4% (v/v) 2-mercaptoethanol, 8 mM 4-(2-aminoethyl)benzenesulfonyl fluoride, and 0.02% (w/v) bromphenol blue). Samples were boiled at 100°C for 10 min, centrifuged at 16,100 *g* for 1 min, before being run on a 12% (w/v) NuPAGE gel at 200V for 55 min with MOPs running buffer and SeeBlue Prestained Molecular Weight Marker. They were then transferred to a PVDF membrane at 35V for 120 min. Following blocking in PBS with 0.05% Tween-20 (PBST) with 5% (w/v) non-fat dried skimmed milk powder, membranes were probed with ICSM35 anti-PrP antibody (1:5000; RML) in PBST overnight. Following washing, the membranes were incubated with a 1:10,000 dilution of alkaline-phosphatase-conjugated goat anti-mouse IgG secondary antibody (A2179) in PBST. After washing (1 h with PBST followed by 2 × 5 min with 20 mM Tris pH 9.8 containing 1 mM MgCl_2_), blots were incubated for 5 min in chemiluminescent substrate (CDP-Star) and visualized on Amersham Hyperfilm. Alternatively, 20% (w/v) brain homogenates were digested with 100 µg/ml pronase for 40 min at 37°C at 1000 rpm. Digestion was terminated with 100 mM EDTA with western blotting being performed as described above.

**Cell-Based Assessment of Infectivity of Infected Brain Homogenates**

Infectivity in brain homogenates were assessed in either the automated scrapie cell assay (ASCA) or the SCA in endpoint format (SCEPA) with the exception of the excluded animals and the clinical endpoint group where animals were not age-matched.

*Automated SCEPA*

The infectivity of brain homogenates prepared from animals culled at 30 dpi, 50 dpi and 70 dpi were assessed by SCEPA as previously described[11], with the research team blinded to sample identity. In brief, an N2a-derived cell line (PK1/2 subclone) was plated in 96-well plates at 15,000 cells/well. The following day the cells were infected with 1 x 10^-4^ or 1 x 10^-6^ concentrations of 10% (w/v) brain homogenate (24 technical replicates/sample/dilution) diluted in OFBS using a Biomek FX liquid handling robot. Three days post infection, cells were split 1:3 and grown to confluence over a 2-day period. After two further 1:3 splits, cells underwent four 1:8 splits over 3-4-day periods with ELISpot plates being prepared at post-split 5 and 6. Here, ∼25,000 cells/well were transferred to activated ELISpot plates, prior to PK treatment and development using the ICSM18 anti-PrP monoclonal antibody followed by an alkaline-phosphatase–conjugated anti-IgG1 polyclonal secondary antibody. The Bioreader 5000-Eβ system was used to quantify PK-resistant PrP-positive cells.

Infectivity of each sample was calculated from the ratio of positive to negative wells, aiming for a ratio between 30% and 70% for one of the two tested concentrations. Samples with a spot count above a defined cut-off, calculated from the average of the non-infected wells + (3 x standard deviation of non-infected wells) were scored as positive. TCIU/mL were calculated according to the Poisson equation, P_(0)_ = e^-m^, where P_(0)_ is the probability of a well remaining uninfected (i.e. non-infected wells/total number of wells). Therefore, TCIU/mL = m x dilution x 3.3, where m = ln [total wells/empty wells]. Samples with very low ratios at 1 x 10^-6^ and with very high ratios at 1 x 10^-4^ underwent a further round of SCEPA whereby cells were infected with 1 x 10^-5^ concentration of the sample. Any samples with 100% infectivity at 1 x 10^-4^ or 1 x 10^-6^ were repeated in the SCA.

*Automated SCA*

The ASCA was used to assess infectivity in samples expected to be above 10^4.5^ TCIU ml^−1^ in 10% (w/v) brain homogenate (90 dpi, 110 dpi, 130 dpi, 140 dpi and some 70 dpi repetitions). ASCA was performed similarly to SCEPA with the exception that 3 x 10^-5^, 3 x 10^-6^ and 3 x 10^-7^ concentrations of brain homogenate were used for infection and four 1:8 splits were performed with intervals ranging from 3-4 days from the time of infection. Additionally, a reference serial dilution of 10% (w/v) RML brain homogenate of known infectivity titre (I8700, 8.64 logs TCIU ml^-1^) was performed to generate a standard curve to deduce the prion titres of the experimental samples. Samples with no measurable activity were repeated.

*Data Analysis*

Samples with no measurable activity from 70 dpi onwards were excluded from analysis as this was not biologically feasible (3 *Stx6^+/+^* and 6 *Stx6^-/-^* mice). Zero values at 30 dpi and 50 dpi could not be excluded due to the very low titres detected in the positive cases. Therefore, they were assigned with a 3.1 log TCIU, the assay lower limit of detection based on historical data. Curves were fitted using a logistic growth model, Y(1-Y/YM), where Y was the current titre and YM was the maximum titre.

**Cell-Based Assessment of Neurotoxicity of Infected Brain Homogenates**

*Brain Homogenate Triple Extraction*

20% (w/v) brain homogenates were combined from 5 animals/genotype culled at 140 dpi to produce a 10% (w/v) brain homogenate pool in DPBS, which was supplemented with 1x Halt™ protease inhibitor cocktail (EDTA-free). Following a triple extraction method (unpublished), the brain homogenates were centrifuged for 10 min at 20°C at 1000 rpm. The supernatant was diluted in neurobasal media supplemented with 2% (vol/vol) B27, 0.25% (vol/vol) GlutaMAX, and 100 U/mL of penicillin–streptomycin to the final concentration of 5×10^-4^ and 2.5×10^-4^. These were applied to mouse primary cortico-hippocampal cultures prepared from embryonic day 17 FVB mouse brains plated at 8000 cells/well. Primary cells were used at the age of 8-10 days *in vitro*. There were 10 technical replicates per treatment with infected brain homogenates and 4 technical replicates per treatment with uninfected brain homogenates. These were tested in three independent experiments comprising independent cell cultures and independent brain homogenates extracts.

*IncuCyte Live Image Acquisition and NeuroTrack Analysis*

Cells were incubated at 37°C and 5% CO_2_ within an IncuCyte S3 live cell imaging apparatus, with four images per well being acquired with a 20× objective lens in phase contrast from the same well coordinates. Images were subsequently analysed with the NeuroTrack module, IncuCyte S3 v.2018B with the following parameters typically being used: segmentation mode—brightness; segmentation adjustment—1.1; neurite sensitivity—0.35; filtering—best; neurite width—2 µm.

Changes in neurite length (average from 4 images in each well) upon treatment were normalised to the baseline neuronal length acquired for 18 hr before treatment, which was set at 100%. Statistical analysis was performed on normalised neurite length values 12 hr post treatment pooled from three independent experiments.

**Biomarker Assessment**

Serum NfL levels were measured at 110 and 140 dpi in singulate using the single-molecule-array (Simoa) NF-light V2 Advantage Kit according to the manufacturer’s instructions. This was run on the Simoa HD-X platform with the experimenter’s blind to study design. Samples with no infectivity as determined by the ASCA/IHC were excluded from the final analysis.

**RNA-Sequencing**

~50 mg cortical brain tissue from uninfected *Stx6^+/+^* and *Stx6^-/-^* mice (5 biological replicates/genotype) was homogenised in 900 μL DNA/RNA Shield in 2.0 mm Bashing Bead Lysis tubes using Precellys 24 (2 x 30 sec at 5500 rpm). Following a 5 min incubation on ice, samples were centrifuged at 16,000 *g* for 1 min with 300 μL of the cleared lysate subsequently being mixed with 300 μL TRI reagent in a RNAase-free tube before addition of 600 μL 98% ethanol. RNA was subsequently extracted using the Directzol RNA Miniprep kit according to the manufacturer’s instructions including an on-column DNase step. An additional DNase digestion and RNA purification procedure was subsequently performed using the RNA Clean and Concentrator kit according to the manufacturer's instructions.

Quality control was performed on the Agilent Technologies 2200 Tape Station using the high sensitivity RNA reagents and on the NanoDrop ND-1000 Spectrometer as per the manufacturer's instructions. One *Stx6*^+/+^ sample was discarded due to poor RNA integrity. 90 ng of total RNA (RIN^e^: >7.9) was used with the Universal Plus Total RNA-Seq with Mouse AnyDeplete kit with library preparation being conducted according to the manufacturer’s instructions. Resulting libraries were visualized on the TapeStation to assess integrity using a D1000 ScreenTape and reagents, according to the manufacturer’s instructions.

Libraries were diluted to 10 nM and then pooled to 4 nM using Qiagen elution buffer. The pool was subsequently diluted to 800 pM with resuspension buffer Tween 20, which was sequenced using the Illumina NextSeq 2000 Kit P2 100 cycles (sequencing configuration: 61-8-8-61) at a loading concentration of 800pM after the addition of 2% PhiX sequencing control library. The data was demultiplexed and converted into a FASTQ file using BCLconvert v3.7.5. Data was analysed using the R software[17] and Bioconductor[5] packages including DESeq2[1, 12] and the SARTools R package[21].

**Prion-Based Cellular Work**

**Generation of Cell Lines with Manipulated *Stx6* Expression**

Cells were routinely split 1:6 every 3 days or 1:8 every 4 days and kept in an incubator at 37°C/5% CO_2._ CAD5 cells were cultured in Opti-MEM + 10% Bovine Growth Serum (BGS) + 1% penicillin-streptomycin (PS) (OBGS). PK1 cells were cultured in Opti-MEM + 10% Fetal Bovine Serum (FBS) + 1% PS (OFBS).

*PiggyBac Transgenesis*

500,000 cells/well were seeded into a 6-well plate in 2 mL media. The following day, a premix containing the PiggyBac transposon vector and PiggyBac transposase vector (mCherry-CAG-hyPBase, 1 µg), 175 μL Opti-MEM and 3.5 μL PLUS reagent was added to 150 μl Opti-MEM with 6 μl Lipofectamine LTX Reagent. After a 5-10 min incubation period at room temperature (RT), 250 μL of this transfection mix was added to the cells. The following day the cells were placed under puromycin selection (2 μg/mL for CAD5 cells; 3 μg/mL for PK1 cells; 4 μg/mL for iS7 cells) with media exchanges every 2-3 days.

*Stable Transfection*

Transfection was performed as described above with the exclusion of the PiggyBac transposase vector. One day post-transfection, the cells were expanded to a 10 cm dish before being placed under antibiotic selection the following day. Media exchanges were performed every 2-3 days for approximately 2-4 weeks until the appearance of distinct colonies of cells. For cell pools, these colonies were pooled into a single suspension and propagated as a cell population. For single cell clones, individual clones were picked and expanded independently to provide a genetically homogenous and clonal expansion.

**Western Blotting**

*Cell Lysate Preparation*

For syntaxin-6 and PrP^C^ western blotting, cell suspensions were harvested by centrifugation at 500 *g* for 4 min with the cell pellet being subsequently washed in ice-cold DPBS. Pellets were lysed in RIPA buffer supplemented with 1x HALT protease inhibitor. Following a 10 min incubation on ice with frequent vortexing, the cell lysates were centrifuged at 16 000 *g* for 10 min at 4˚C. The supernatant was collected and stored at -20˚C until use.

For abnormal PrP, cells were collected from each well of a 6-well plate in 1 mL ice-cold DPBS on ice and spun at 300 *g* for 4 min at 4°C. The pellet was lysed in RIPA buffer supplemented with 4 µL/ml benzonase on ice with gentle agitation employed every 5 min for 20 min.

*Immunoblotting*

For total protein concentration estimation, Pierce™ BCA Protein Assay kit was used according to the manufacturer’s instructions. Lysates were diluted in DPBS and 4x Laemelli sample buffer to obtain a final 1x concentration with 355 mM final 2-mercaptoethanol. Following boiling at 95°C for 5 min, samples were loaded onto a 4-12% Bis-Tris polyacrylamide gel in addition to the SeeBlue Protein ladder. Following electrophoresis at 180V for 1 hr, protein was electroblotted onto a nitrocellulose membrane at 35 V for 2 hr. Following a 1 hr incubation at RT in Odyssey PBS Blocking Buffer with agitation, membranes were probed either with anti-syntaxin-6 (clone C34B2; 1:500) or with monoclonal anti-PrP antibody (clone ICSM18; 1:1,250) overnight at 4°C. 1:1 dilution of Odyssey Blocking Buffer and PBST was used as diluent for all antibody incubation steps.

For syntaxin-6 immunodetection, membranes were washed 3x with PBST for 5 min with agitation, followed by a 1 hr incubation with anti-β actin (ab6276; 1:5000) at RT with agitation. After 3x 5 min washes in PBST, membranes were probed with fluorophore-conjugated secondary antibodies IRDye 800CW Donkey anti-rabbit IgG (1:4000) and IRDye 680RD goat anti-mouse IgG (1:20,000) for 1 hr at RT with agitation and protected from light. Following 3x 5 min washes in PBST, membranes were imaged in PBS using the Odyssey® Imaging System (LI-COR; Model 9120).

Revert^TM^ 700 Total Protein Stain was used for normalisation for some syntaxin-6 western blots, which was used according to the manufacturer’s instructions. Membranes were imaged in the 700 nm channel before being blocked and treated as described above excluding the β-actin immunostaining.

For PrP immunodetection, membranes were washed with PBST for 5 min x3 with agitation, followed by a 1 hr incubation with IRDye 680RD goat anti-mouse IgG (1:10,000, LI-COR) at RT with agitation protected from light. After 3x 5 min washes in PBST, membranes were imaged in PBS using the Odyssey Reader System. Membranes were subsequently incubated with rabbit anti-β actin (A2066; 1:1000). Following 3x 5 min washes in PBST, membranes were probed with IRDye 800CW Donkey anti-rabbit IgG (1:10,000) for 1 hr at RT with agitation. Following 3x 5 min washes in PBST, membranes were imaged in PBS.

Abnormal PrP western blotting was performed as described previously with gel electrophoresis being performed using 12% NuPAGE gels run at 200 V for 50 min followed by a 2 hr transfer at 35 V with 6D11 being used as the primary antibody (1:5000, ON).

*Western Blot Analysis*

Image Studio™ Software (licor.com/islite) was used to quantify the fluorescent signals from the 700 nm and 800 nm channels. Rectangles were drawn around the target bands as well as the loading control bands. Median background subtraction method was used. Following quantification, the *Lane Normalisation Factor* (LNF) was calculated as follows:

$$LNF=\frac{\beta-Actin Signal for Each Lane}{\beta-Actin Signal from Lane with Highest \beta-Actin Signal}$$

The normalised signal was then calculated using the following formula:

$$Normalised Signal=\frac{Target Signal for Each Lane}{LNF for Each Lane}$$

**Growth Rate Analysis**

3.5 x 10^5^ cells were seeded in a T25 flask with cell doubling time being calculated after 4 days using the following equation, where N_4_= cell number on day 4 and N_0_ = 3.5 x 10^5^.

$$Doubling time \left( days \right)= \frac{4}{\log_{2} \frac{N_{4}}{N_{0}}}$$

**Scrapie Cell Assay (SCA)**

*Automated Scrapie Cell Assay*

The ASCA was performed as previously described[11] with some modifications. Cells were counted using a C-CHIP disposable haemocytometer and subsequently seeded at 18,000 cells/well in a 96-well plate. The following day, the cells were infected with a dilution series of 10% (w/v) prion-infected brain homogenate. Uninfected cells were also included to provide an estimation of the background spot count. Cells were passaged 1:6 every 3 days or 1:8 every 4 days, using the automated Biomek FX liquid handling robot.

After a set number of passages, 85 μL of the 270 μL cell suspension was plated on ELISpot IP Filter Plates (PVDF membrane, 0.45 μm), which had been activated with 30% (v/v) ethanol. Following two PBS washes, the membranes were dried at 50°C. Plates were subsequently treated with proteinase K (1:10,000) in lysis buffer (50 mM Tris HCl pH 8, 150 mM NaCl, 0.5% (w/v) sodium deoxycholate, 0.5% (v/v) Triton X-100) at 37°C for 1 hr. Plates were washed twice in PBS before being treated with 3M guanidine thiocyanate in 10 mM Tris.HCl (pH 8) and incubated at RT for 20 min for decontamination and antigen retrieval. Following seven PBS washes, plates were incubated with 0.5x SuperBlock TBS blocking buffer for 30 min at RT.

For immunodetection, plates were incubated with an anti-PrP antibody (clone ICSM18; D-Gen Ltd; 0.4 μg/mL) in Tris Buffered Saline with Tween 20 (TBST)/1% milk power at RT for 1 hr. After 5 washes with TBST, plates were incubated with an alkaline phosphatase-linked anti-mouse secondary antibody (IgG1-AP; 1:8000) in TBST/1% milk power at RT for 1 hr. Following 5 further washes in TBST, the plates were dried and treated with the alkaline phosphatase conjugate substrate and incubated for 30-60 min at RT to allow visualisation of the spots. Following two washes with ddH_2_O, plates were dried and the number of PK-resistant PrP infected cells were counted using the Bioreader 5000-Eβ.

For the secreted infectivity of iS7 cells with syntaxin-6 manipulation, 100 µL of 1:10 dilution of cells in DPBS was plated on ELISpot. For assessment of PK1 *Stx6* OE *de novo* infection secreted infectivity, 85 μL of the 270 μL cell suspension was plated on ELISpot.

*Manual SCA*

For infection, a cell suspension (50,000 cells/mL) was prepared with 3 µl exosome-enriched cell supernatants/mL (prepared from iS7 cells as described in[19]) with 300 µl plated/well in a 96-well plate for 3-4 days. Cells were subsequently split 1:5 or 1:6 every 3-4 days. For chronically infected cells, 300 µl cells (50,000 cells/mL) were plated in 96-well plates with ELISpot assessment of prion steady state levels being conducted across three passage numbers.

The SCA was performed as above with the following modifications: ELISpot plates were activated in 50% ethanol, blocking was performed for 1 hr, the primary antibody employed was 6D11 (1:10,000) and 5x TBS-T washes were conducted following the secondary antibody incubation.

*Haematoxylin Staining*

Haematoxylin was filtered with a 0.22 µm filter before being diluted 1:10 in H_2_O. 100 µL/well was added for 1 min to the ELISpot plate (post-immunostaining) followed by two washes with H_2_O. Plates were subsequently read using the Bioreader 5000-Eβ reader with high sensitivity parameters. The haematoxylin total cell count was used to normalise the raw spot count providing an estimation of the proportion of infected cells in assays where the cell count was not saturated.

**Confocal Microscopy**

500 µL cell suspension (50,000 cells/mL) were plated in 8-well chambered glass coverslides and incubated at 37°C/5% CO_2_ for 4 days before fixation for 12 min with 3.7% formaldehyde in PBS. As previously described for PrP^d^[14, 19], the cells were washed once in PBS before a 1 min incubation with chilled acetone. Following one PBS wash, cells were incubated for 10 min with 3.5 M guanidine thiocyanate before five further washes in PBS.

Cells were incubated at 4°C overnight with primary antibodies (Anti-PrP (6D11), 1:10,000; Anti-PrP (5B2), 1:500; Anti-Lamp1 (1D4B). 1:1000; Anti-Eea1 (C45B10), 1:1000; Anti-TGN46 (ab16059), 1:1000) diluted into Superblock (1:4 dilution in PBS/10% PS). After one wash with PBS, cells were labelled with fluorescence-conjugated secondary antibodies (1:1000) and DAPI (1:10,000) and incubated at 4 °C overnight. Following a final wash with PBS, cells were stored in PBS/10% PS at 4°C until imaging.

*Image Acquisition*

A Zeiss LSM710 laser-scanning microscope was used for image acquisition using a ×63 objective (1.4 oil) with Immersol immersion oil. The 460-540 nm and 565–640 nm bandpass filters were employed to measure AlexaFluor488 and Rhodamine Red-X fluorescence, subsequent to excitation with an argon laser at 488 nm and a diode-pumped solid state laser at 561 nm, respectively.

For PK1 *Stx6* knockdown cells infected with exosomes, an automated scan of 7 x 7 tiles, with approximately 25 cells per tile, was set up for each condition (images with <11 cells were excluded). Using Volocity, the signal of 5B2 was digitally increased by 3 iterations of dilation, before quantifying its intersection with 6D11, which was used as a proxy of plasma membrane staining based on[19]. To quantitatively and specifically assess perinuclear PrP^d^, the 6D11 fluorescence intensity and area was determined excluding 6D11 signal touching the intersection with 5B2. For each image, the perinuclear PrP^d^ signal was normalised to the number of cells in the field view as determined by Volocity quantification of the DAPI count. Tile scans were also conducted in chronically infected cells for quantification of the total signal intensity and area of 5B2 and 6D11 staining using Volocity.

To determine levels of colocalisation of 6D11-positive PrP^d^ with intracellular markers in chronically infected cells, ~20 magnified images were acquired, which were subsequently used to determine Pearson’s correlation with Costes threshold correction[4] using Velocity. Negative values were computed as zero. Additionally, the Volocity cropping function was used to manually isolate the perinuclear 6D11-positive PrP^d^ signal which was subsequently quantified in terms of fluorescence intensity and area.

To quantitatively assess morphological differences in fibril-like PrP^d^ aggregates at the plasma membrane, stacks of 10 images in the z-direction above the basement membrane were recorded with a step size of 0.1 µm with stacks subsequently being aggregated into a maximum intensity projection. Length of fibrils were measured using the line tool on Volocity in a blinded fashion.

**Cell Viability Assay**

Cell viability was assessed using the CellTitre-Glo luminescent assay. In brief, cells were split into a CELLSTAR white 96-well plate with a micro-clear bottom. After 3-4 days of growth, media was removed from cells and the reconstituted CellTitre-Glo buffer/substrate mix was added (75 µL/well). On the TECAN Infinite F200 PRO machine, the plate underwent shaking for 2 min followed by a 10 min incubation before luminescence was recorded.

**Flow Cytometry Quantification of Total PrP Levels**

Cells were resuspended and counted on the Countess 3 FL Automated Cell Counter using the live cell count. 0.5 ×10^6^ cells were centrifuged at 1500 rpm for 5 min at 4°C, with these centrifugation settings being used for all further centrifugations. Cells were then resuspended in 250 µL of BD Cytofix/Cytoperm Fixation/Permeabilisation solution for 20 min on ice, followed by two washes 1× BD Perm/Wash™ buffer.

Cells were subsequently incubated with an anti-PrP antibody (ICSM18 subclone, 10 µg/mL) diluted in PBS/1%FBS for 30 min on ice. Following another wash in PBS/1%FBS, the cells were incubated with an AlexaFluor 647-conjugated secondary antibody diluted in PBS/1%FBS for 30 min on ice (1:1000). Following another wash step, the cells were resuspended in 100 μL PBS/1%FBS, which was analysed using a Beckman Coulter CytoFLEX equipped with blue 488nm and red 633nm lasers, with AF647 being detected using the 660/10 filter.

Cells were gated based on the forward and side scatter profiles using forward scatter width to identify single cells. A total of 50,000 events in the single-cell gate were recorded per sample. A secondary only control was included in all experiments to aid gating. Further analyses were carried out in FlowJo software with median fluorescence of anti-IgG-AF647 binding to ICSM18 being used to determine differences in total or cell surface PrP levels.

**RT-qPCR Assessment of *Prnp* RNA Levels**

*Prnp* RNA levels were assessed by performing two-step RT-qPCR according to the manufacturer’s instructions using the Quantitect Reverse Transcription Kit with 2 µl RNA input. cDNA was amplified using QuantiTect Primer Assays against *Prnp* (Mm_Prnp_1_SG) and ActB (Mm_Actb_1_SG) as the loading control.

**Cycloheximide Assay Assessing PrP Degradation Kinetics**

PK1 overexpression cell lines were seeded in 6-well format (0.3 x 10^6^ cells/well). After 48 hr, media was exchanged with fresh media containing 100 µg/mL cycloheximide or DMSO vehicle control. Cells were subsequently harvested at 0 hr, 3 hr and 6 hr (3 replicas/cell line/time point) and immediately fixed in Fixation/Permeabilization solution. The following day, total PrP levels were assessed by flow cytometry as described previously**.** The initial fluorescence at 0 hr was used for normalisation of each cell line to monitor relative protein decay over time.

**Assessment of Secreted Infectivity**

*iS7 Cells with Transient Stx6 Knockdown*

iS7 cells (1.33 x 10^5^ cells/mL) were reverse transfected with pools of 30 custom-designed siRNAs (siPools)[8] against *Stx6* or a non-silencing control. In brief the siPools were prepared at a stock concentration of 10 µM in nuclease-free water. 2 µL siPool and 6 µL RNAimax transfection reagent were incubated with 42 µL Opti-MEM for a 5 min incubation. 950 µL OFBS was subsequently added to the mix. 150 µL was then added to each well of a 96-well plate (12 wells/condition) followed by 150 µL cell suspension and left to grow for 3 days. On day 3, the media was replaced for a 7-hr incubation. Media from the 12 wells/condition was pooled, clarified at 10,000 *g* for 10 min and stored in the fridge until the time of infection. Cells were collected and washed in DPBS to confirm successful knockdown by immunoblotting as described previously.

*PK1 Cell Lines with Stable Syntaxin-6 Manipulation*

For secreted infectivity assessment following acute infection of PK1 cell lines with stable *Stx6* manipulation, after 3-4 days of growth, the conditioned media from 3 wells of a 6 well plate/cell line was harvested. This was clarified at 4,700 *g* for 10 min and stored at -20°C until the time of infection.

*iS7 Cell Lines with Stable Syntaxin-6 Manipulation*

iS7 cells (180,000 cells/mL) were seeded in a 12-well plate (4 wells/cell line) and left to grow for 4 days. On day 4, the media was replaced with 1 mL fresh media/well. Following a 6 hr incubation, the conditioned media was collected and pooled per cell line and subsequently clarified at 10,000 *g* for 10 min and stored this at -20°C until the time of infection.

*Infection*

The clarified supernatant was used to infect reporter PK1 cells that had been seeded the day before with the SCA being conducted as described previously with cells being diluted 1:10 prior to plating on ELISpot across four splits.

**Key Resources Table**

| REAGENT or RESOURCE | SOURCE | IDENTIFIER |
| --- | --- | --- |
| Antibodies | | |
| Anti-Syntaxin 6 Antibody [C34B2] | Cell Signalling Technologies | Cat#2869; RRID:AB_2196500 |
| Anti-PrP Antibody [ICSM18] | D-Gen Ltd | PABC-107 |
| Anti-PrP Antibody [ICSM35] | D-Gen Ltd | NA |
| Anti-PrP Antibody [6D11] | Biolegend | 808001; RRID:AB_2564735 |
| Anti- PrP Antibody [5B2] | Santa Cruz | sc-47730; RRID:AB_628170 |
| Anti-β-Actin Antibody [AC-15] | Abcam | Ab6276; RRID:AB_2223210 |
| Anti-β-Actin Antibody [AC-15] | Sigma | A5441; RRID:AB_476744 |
| Anti-β-Actin Antibody | Sigma | A2066; RRID:AB_476693 |
| Anti-Lamp1 Antibody [1D4B] | Santa Cruz Biotechnology | sc-19992; RRID:AB_2134495 |
| Anti-Eea1 Antibody [C45B10] | Cell Signaling Technology | Cat#3288; RRID:AB_2096811 |
| Anti-TGN46 Antibody | Abcam | ab16059; RRID:AB_443307 |
| Anti-Iba1 Antibody | AlphaLaboratories | Cat#019-19741; RRID:AB_839504 |
| Anti-GFAP Antibody | Dako, Agilent | Z0334; RRID:AB_10013382 |
| Anti-Synaptophysin Antibody | Invitrogen | PA5-27286; RRID:AB_2544762 |
| Goat Anti-rabbit secondary antibody | Abcam | Ab6720; RRID:AB_954902 |
| Alkaline Phosphatase conjugated anti-mouse IgG | Sigma | A2179; RRID:AB_257981 |
| Alkaline Phosphatase anti-IgG | Southern Biotechnology | 1010-04; RRID:AB_2794124 |
| IRDye 800CW donkey anti-rabbit | LI-COR | 926-32213; RRID:AB_621848 |
| IRDye 680RD goat anti-mouse IgG | LI-COR | 926-68070; RRID:AB_10956588 |
| Cross-adsorbed anti-mouse H+L-AF488 | Jackson, Stratech | 115-545-166-JIR; RRID:AB_2338852 |
| Cross-adsorbed anti-mouse H+L-Rhodamine X | Jackson, Stratech | 115-295-166-JIR; RRID:AB_2338768 |
| Cross-adsorbed anti-mouse IgG1-AF488 | Jackson, Stratech | 115-545-205-JIR; RRID:AB_2338854 |
| Cross-adsorbed anti-mouse IgG2a-Rhodamine X | Jackson, Stratech | 115-295-206-JIR; RRID:AB_2338770 |
| Cross-adsorbed anti-mouse IgG2b Rhodamine X | Jackson, Stratech | 115-295-207-JIR; RRID:AB_2338771 |
| Cross-adsorbed anti-mouse IgG1 AlexaFluor647 | Jackson, Stratech | 115-505-205-JIR |
| Cross-adsorbed anti-rabbit H+L-AF488 | Jackson, Stratech | 111-545-144-JIR; RRID:AB_2338052 |
| Cross-adsorbed anti-rabbit IgG TRITC | Jackson, Stratech | 711-025-152-JIR; RRID:AB_2340588 |
| Cross-adsorbed anti-rat IgG Rhodamine | Jackson, Stratech | 712-295-150-JIR; RRID:AB_2340675 |
| Biological samples | | |
| RML-Infected CD1 Brain Homogenate | Laboratory of Jonathan Wadsworth | I8700 |
| 22L-Infected C57BL/6 Brain Homogenate | Laboratory of Jonathan Wadsworth | I23185 |
| MRC2-Infected SJL Brain Homogenate | Laboratory of Jonathan Wadsworth | I17800 |
| ME7-Infected C57BL/6 Brain Homogenate | Laboratory of Jonathan Wadsworth | I21487 |
| Exosome Fraction from Infected Cells | Laboratory of Peter Klöhn (Described in Ribes *et al.* (2023)[19]) | NA |
| RML-Infected C57BL/6 Brain Homogenate | Laboratory of Jonathan Wadsworth | I21488 |
| Normal C57BL/6 Brain Homogenate | Laboratory of Jonathan Wadsworth | 14052 |
| Chemicals, peptides, and recombinant proteins | | |
| Superblock Protein in 2.5 mM Tris, 0.15 M NaCl, pH 7.4 | Thermo Scientific | 0037545 |
| Lipofectamine™ LTX Reagent with PLUS™ Reagent | Thermo Fisher | A12621 |
| Cycloheximide solution | Sigma | C4859 |
| RIPA Buffer | Sigma | R0278 |
| Halt™ Protease Inhibitor Cocktail, EDTA-free (100X) | ThermoFisher Scientific | 87785 |
| Benzonase | EMD Millipore | 70664 |
| Proteinase K | Roche | 3115828001 |
| Pronase (Protease from *Streptomyces griseus*) | Sigma | P5147 |
| DAB Map Detection Kit | Roche Tissue Diagnostics | 05266360001, 760-124 |
| CellTiter-Glo® Reagent | Promega | G7570 |
| Critical commercial assays | | |
| Cytofix/Cytoperm™ Fixation/Permeabilization Kit | BD Biosciences | 554714 |
| Pierce BCA Protein Assay Kit | Thermo Scientific | 23227 |
| NF-light V2 Advantage Kit | Quanterix | 104073 |
| Universal Plus Total RNA-Seq with Mouse AnyDeplete kit | Tecan | 9157-A01 |
| NextSeq 2000 Kit P2 100 cycles | Illumina | NA |
| Experimental models: Cell lines | | |
| Mouse: PK1/2 Cell Line (Derivative of Mouse Neuro2a Cells, ATCC, CCL-131) | Laboratory of Peter Klöhn (Described in Klöhn *et al.* (2003)[11]) | NA |
| Mouse: S7 (Derivative of N2aPK1 Cells) | Laboratory of Peter Klöhn (Described in Marbiah *et al.* (2014)[14]) | NA |
| Mouse: iS7 Cells (Persistently Infected S7 Cells) | Laboratory of Peter Klöhn (Described in Ribes *et al.* (2023)[19]) | NA |
| Mouse: CAD-2A2D5 (CAD5) Cells (Derivative from Cath.a-differentiated (CAD) Cells Described in Qi *et al.* (1997)[16] | Laboratory of Charles Weissman (Described in Mahal *et al.* (2014)[13]) | NA |
| Experimental models: Organisms/strains | | |
| Mouse: C57BL/6NTac-Stx6^em1(IMPC)H^ | UKRI-MRC Harwell as part of the IMPC () | Allele MGI ID: 6266827; EM:12503 |
| Oligonucleotides | | |
| Primers for PCR Genotyping of *Stx6^+/+^*, *Stx6^+/-^* and *Stx6^-/-^* mice: CGATCTGTGAGACTCATCGGG, GGGAGTCCTAACACCACCTTC, GGACACCATGCTTTCAAGATTT. | Jones *et al.* (2024)[10] | NA |
| shRNA targeting sequence: shSTX6_A: CAGATGTCAGCTTCATCTGTGCAGGCACT | Insight Biotechnology | NA |
| shRNA targeting sequence: shSTX6_B: GGAGAGGTACAGAAAGCAGTCAACACTGC | Insight Biotechnology | NA |
| shRNA targeting sequence: shSTX6_C: CCAGTGGTGTGCCATAGCCATCCTCTTCG | Insight Biotechnology | NA |
| shRNA targeting sequence: shSTX6_D: TGGAATGCTGGAGTGGCAGATCGCTATGG | Insight Biotechnology | NA |
| shRNA targeting sequence: shNSC: GCACTACCAGAGCTAACTCAGATAGTACT | Insight Biotechnology | NA |
| shRNA targeting sequence: PRNP (Si8): GAGACAATCTAAACATTCT | Goold et al. (2011)[6] | NA |
| Recombinant DNA | | |
| pGFP-V-RS shSTX6_A, B, C & D and shNSC | Insight Biotechnology | TG517176A, TG517176B, TG517176C, TG517176D, TR30013 |
| pRetroSuper containing Prnp-targeting shRNA (Si8) | Laboratory of Parmjit Jat (Originally Described in Goold et al. (2011)[6]) | NA |
| pRP[Exp]-mCherry-CAG>hyPBase | VectorBuilder | VB010000-9365tax |
| pPB[shRNA]-EGFP:T2A:Puro-U6>(mStx6[shRNA#4]) | VectorBuilder | VB221229-1281dfp |
| pPB[shRNA]-EGFP:T2A:Puro-U6>(NSC[shRNA]) | VectorBuilder | VB230103-1034jjw |
| pPB[shRNA]-EGFP:T2A:Puro-U6>(Prnp[shRNA]) | VectorBuilder | VB230103-1039ctx |
| pPB[Exp]-EGFP/Puro-mPGK>mStx6[NM_021433.3] | VectorBuilder | VB220821-1010eff |
| pPB[Exp]-EGFP/Puro-mPGK>ORF_Stuffer | VectorBuilder | VB220828-1137yrp |
| Software and algorithms | | |
| ImageLab 6.0 | Biorad | https://www.bio-rad.com/en-uk/product/image-lab-software |
| NDP.serve3 | NanoZoomer Digital Pathology | https://nanozoomer.unisa.edu.au/ndp/serve/home |
| NZConnect 1.0.36 (IVD) | Hamamatsu | https://nzconnect.ion.ucl.ac.uk/nz/connect/home |
| Qupath (Version 0.4.3) | Bankhead et al.[2] | https://qupath.github.io/) |
| i-control (Version 3.4.2.0) | Tecan | NA |
| Odyssey Acquisition Software (Version 2.1) | LI-COR | https://www.licor.com/bio/las/ |
| Image Studio Lite Ver 5.2 | LI-COR | Discontinued |
| ZEN 2.3 SP1 (Black Edition) | Zeiss | https://www.micro-shop.zeiss.com/en/us/softwarefinder/software-categories/zen-black/zen-black-system/ |
| ZEN 2.5 (Blue Edition) | Zeiss | https://www.micro-shop.zeiss.com/en/de/softwarefinder/software-categories/zen-blue/ |
| ImageJ 1.53n | National Institutes of Health, USA | NA |
| InVivoStat v4.7.0 | Clark et al. (2012)[3] | https://invivostat.co.uk/download/ |
| GraphPad Prism 9 | GraphPad | https://www.graphpad.com/features |
| Incucyte 2022B Rev2 | Satorius | https://downloads.essenbioscience.com/get/incucyte-2022b-rev2-gui |
| Tapestation Analysis Software A.02.02 (SP1) | Agilent | NA |

**References**

1 Anders S, Huber W (2010) Differential expression analysis for sequence count data. Genome Biology 11: R106 Doi 10.1186/gb-2010-11-10-r106

2 Bankhead P, Loughrey MB, Fernández JA, Dombrowski Y, McArt DG, Dunne PD, McQuaid S, Gray RT, Murray LJ, Coleman HGet al (2017) QuPath: Open source software for digital pathology image analysis. Scientific Reports 7: 16878 Doi 10.1038/s41598-017-17204-5

3 Clark RA, Shoaib M, Hewitt KN, Stanford SC, Bate ST (2012) A comparison of InVivoStat with other statistical software packages for analysis of data generated from animal experiments. J Psychopharmacol 26: 1136-1142 Doi 10.1177/0269881111420313

4 Costes SV, Daelemans D, Cho EH, Dobbin Z, Pavlakis G, Lockett S (2004) Automatic and Quantitative Measurement of Protein-Protein Colocalization in Live Cells. Biophys J 86: 3993-4003 Doi <https://doi.org/10.1529/biophysj.103.038422>

5 Gentleman RC, Carey VJ, Bates DM, Bolstad B, Dettling M, Dudoit S, Ellis B, Gautier L, Ge Y, Gentry Jet al (2004) Bioconductor: open software development for computational biology and bioinformatics. Genome Biology 5: R80 Doi 10.1186/gb-2004-5-10-r80

6 Goold R, Rabbanian S, Sutton L, Andre R, Arora P, Moonga J, Clarke AR, Schiavo G, Jat P, Collinge Jet al (2011) Rapid cell-surface prion protein conversion revealed using a novel cell system. Nature Communications 2: 281 Doi 10.1038/ncomms1282

7 Hamilton MA, Russo RC, Thurston RV (1977) Trimmed Spearman-Karber method for estimating median lethal concentrations in toxicity bioassays. Environmental Science & Technology 11: 714-719 Doi 10.1021/es60130a004

8 Hannus M, Beitzinger M, Engelmann JC, Weickert MT, Spang R, Hannus S, Meister G (2014) siPools: highly complex but accurately defined siRNA pools eliminate off-target effects. Nucleic Acids Res 42: 8049-8061 Doi 10.1093/nar/gku480

9 Jones E, Hill E, Linehan J, Nazari T, Caulder A, Codner GF, Hutchison M, Mackenzie M, De Oliveira MW, Al-Doujaily Het al (2023) Knockout of Sporadic Creutzfeldt-Jakob Disease Risk Gene in Mice Extends Prion Disease Incubation Time. bioRxiv: 2023.2001.2010.523281 Doi 10.1101/2023.01.10.523281

10 Jones E, Hill E, Linehan J, Nazari T, Caulder A, Codner GF, Hutchison M, Mackenzie M, Farmer M, Coysh Tet al (2024) Characterisation and prion transmission study in mice with genetic reduction of sporadic Creutzfeldt-Jakob disease risk gene Stx6. Neurobiology of disease 190: 106363 Doi <https://doi.org/10.1016/j.nbd.2023.106363>

11 Klöhn PC, Stoltze L, Flechsig E, Enari M, Weissmann C (2003) A quantitative, highly sensitive cell-based infectivity assay for mouse scrapie prions. Proceedings of the National Academy of Sciences of the United States of America 100: 11666-11671 Doi 10.1073/pnas.1834432100

12 Love MI, Huber W, Anders S (2014) Moderated estimation of fold change and dispersion for RNA-seq data with DESeq2. Genome Biology 15: 550 Doi 10.1186/s13059-014-0550-8

13 Mahal SP, Baker CA, Demczyk CA, Smith EW, Julius C, Weissmann C (2007) Prion strain discrimination in cell culture: the cell panel assay. Proceedings of the National Academy of Sciences of the United States of America 104: 20908-20913 Doi 10.1073/pnas.0710054104

14 Marbiah MM, Harvey A, West BT, Louzolo A, Banerjee P, Alden J, Grigoriadis A, Hummerich H, Kan HM, Cai Yet al (2014) Identification of a gene regulatory network associated with prion replication. The EMBO journal 33: 1527-1547 Doi 10.15252/embj.201387150

15 O'Shea M, Maytham EG, Linehan JM, Brandner S, Collinge J, Lloyd SE (2008) Investigation of mcp1 as a quantitative trait gene for prion disease incubation time in mouse. Genetics 180: 559-566 Doi 10.1534/genetics.108.090894

16 Qi Y, Wang JK, McMillian M, Chikaraishi DM (1997) Characterization of a CNS cell line, CAD, in which morphological differentiation is initiated by serum deprivation. The Journal of neuroscience : the official journal of the Society for Neuroscience 17: 1217-1225 Doi 10.1523/jneurosci.17-04-01217.1997

17 R Core Team (2022) R: A Language and Environment for Statistical Computing. City

18 Rangel A, Race B, Phillips K, Striebel J, Kurtz N, Chesebro B (2014) Distinct patterns of spread of prion infection in brains of mice expressing anchorless or anchored forms of prion protein. Acta Neuropathol Commun 2: 8 Doi 10.1186/2051-5960-2-8

19 Ribes JM, Patel MP, Halim HA, Berretta A, Tooze SA, Klöhn P-C (2023) Prion protein conversion at two distinct cellular sites precedes fibrillisation. Nature Communications 14: 8354 Doi 10.1038/s41467-023-43961-1

20 Sandberg MK, Al-Doujaily H, Sharps B, De Oliveira MW, Schmidt C, Richard-Londt A, Lyall S, Linehan JM, Brandner S, Wadsworth JDFet al (2014) Prion neuropathology follows the accumulation of alternate prion protein isoforms after infective titre has peaked. Nature Communications 5: 4347 Doi 10.1038/ncomms5347

21 Varet H, Brillet-Guéguen L, Coppée J-Y, Dillies M-A (2016) SARTools: A DESeq2- and EdgeR-Based R Pipeline for Comprehensive Differential Analysis of RNA-Seq Data. PloS one 11: e0157022 Doi 10.1371/journal.pone.0157022

22 Wadsworth JD, Asante EA, Desbruslais M, Linehan JM, Joiner S, Gowland I, Welch J, Stone L, Lloyd SE, Hill AFet al (2004) Human prion protein with valine 129 prevents expression of variant CJD phenotype. Science (New York, NY) 306: 1793-1796 Doi 10.1126/science.1103932
