# Supplementary material for: Intracellular trafficking SNARE protein, syntaxin-6, modifies prion cellular phenotypes and risk of disease development in vivo
Source: Acta Neuropathol. 2025 Nov 4;150(1):48. doi: 10.1007/s00401-025-02946-8 (PMC12586215; doi:10.1007/s00401-025-02946-8)
Supplement: Supplementary file 2 — Supplementary file2: Supplementary data (DOCX 9960 KB) [file 401_2025_2946_MOESM2_ESM.docx]

# Supplementary Figures


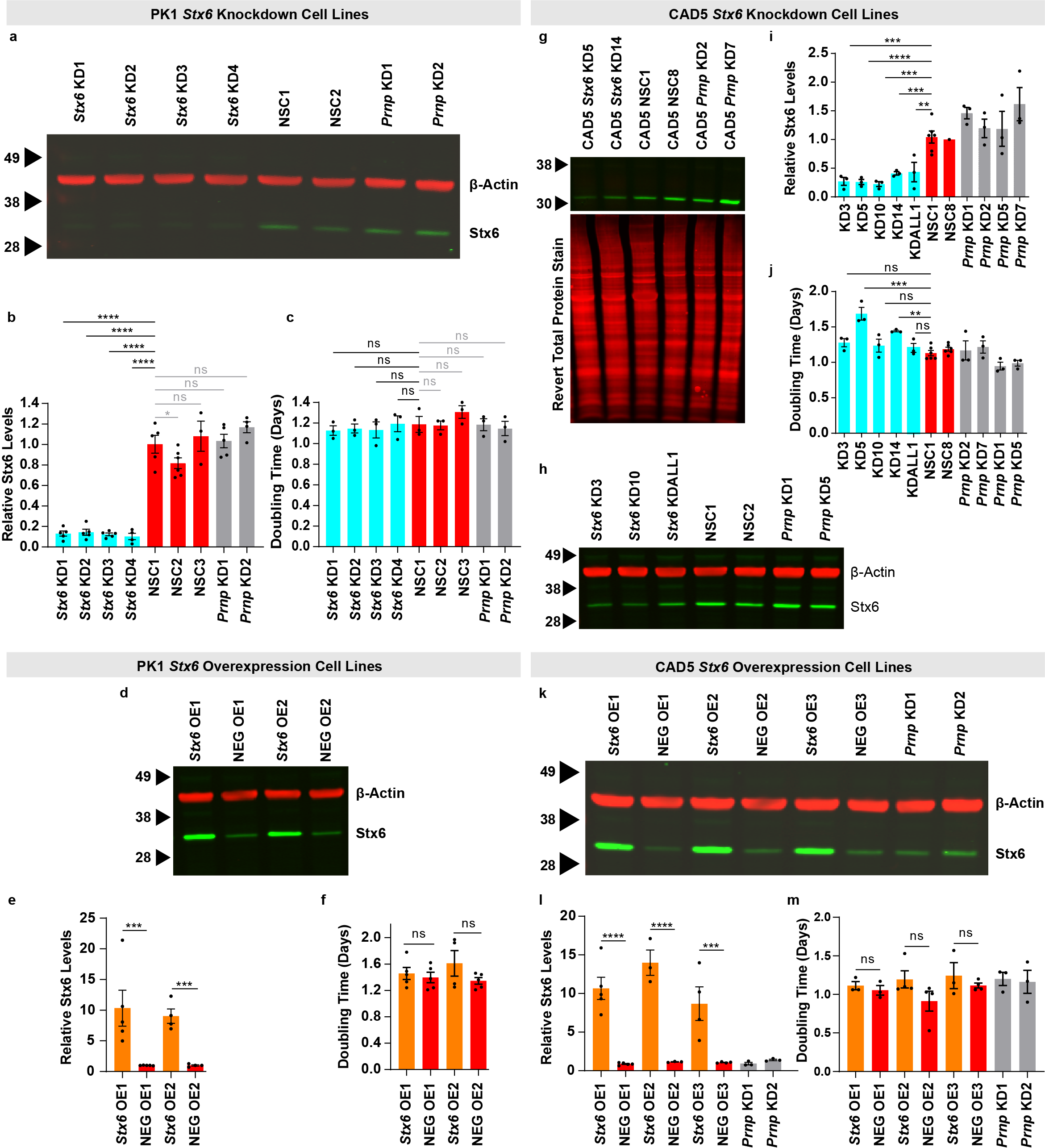


### Supplementary Figure 1 Characterisation of Prion Susceptible Cell lines with Syntaxin-6 Manipulation. Related to Figure 1. (a) Immunoblot probing for syntaxin-6 levels (bottom, green) and β-Actin (top, red) in PK1 cells stably expressing shRNAs targeting *Stx6* or *Prnp* or shRNAs encoding a non-silencing control scrambled sequence (NSC1-2). (b) Quantification of syntaxin-6 band intensity relative to the β-Actin loading control followed by normalisation to the average normalised signal of the NSC and *Prnp* knockdown cell lines. Bars show mean ± SEM. Each dot represents a different biological replicate with cells being harvested at different passage numbers to confirm stable knockdown. Statistical differences were assessed using one-way ANOVA followed by Fisher’s LSD test assessing differences of all cell lines to NSC1. (c) Growth rate measured as the doubling time in days (n=3 biological replicas). Bars and error bars: mean ± SEM. Significance levels based on one-way ANOVA with Fisher’s LSD post-hoc test comparing to NSC1. (d) Immunoblot probing for syntaxin-6 levels and β-Actin in PK1 cells which have either been stably transfected with a vector encoding the *Stx6* open reading frame (*Stx6* OE) or a vector encoding an unrelated sequence (NEG OE). (e) Quantification of syntaxin-6 band intensity normalised to the β-Actin loading control followed by normalisation to the corresponding negative control line. Bars show mean ± SEM. Following rank transformation, statistical differences were assessed using one-way ANOVA followed by the pre-planned comparisons of matched cell lines. (f) Growth rate measured as the doubling time in days (n=3-4 biological replicas). Bars and error bars: mean ± SEM. Significance levels based on one-way ANOVA with Fisher’s LSD post-hoc test of pre-planned comparisons of matched cell lines. (g,h) Representative immunoblot for syntaxin-6 levels as well as either total protein stain (g) or β-Actin (h) in CAD5 cells stably expressing shRNAs targeting *Stx6* or *Prnp* or shRNAs encoding a NSC sequence. (i) Quantification of syntaxin-6 band intensity normalised to either total protein levels or β-Actin, relative to NSC8. Statistical differences were assessed using one-way ANOVA followed by Fisher’s LSD test assessing differences of all cell lines to NSC1. (j) ­­Growth rate measured as the doubling time in days (n=3 biological replicas). Bars and error bars: mean ± SEM. As the assessment was conducted across three batches, batch 2 and 3 values were normalised to NSC1 in batch 1 for visual comparability. Significance levels based on one-way ANOVA with Fisher’s LSD post-hoc test of pre-planned comparisons with each batch being assessed separately. (k) Immunoblot for syntaxin-6 levels and β-Actin in CAD5 cells which had either been stably transfected with a vector encoding the *Stx6* open reading frame (*Stx6* OE) or a vector encoding an unrelated sequence (NEG OE). (l) Quantification of syntaxin-6 band intensity normalised to the β-Actin loading control followed by normalisation to the corresponding negative control line. Bars show mean ± SEM. Statistical differences were assessed using one-way ANOVA followed by pre-planned comparisons of matched cell lines. (m) Growth rate measured as doubling time in days (n=3-4 biological replicas). Bars and error bars: mean ± SEM. Significance levels based on one-way ANOVA with Fisher’s LSD post-hoc test of pre-planned comparisons of matched cell lines. Blots in (D), (G) and (H) were cropped to contain only relevant image data. Brightness and contrast of the western blots were optimally adjusted. *P < 0.05, **P < 0.01, ***P < 0.001, ****P < 0.0001.


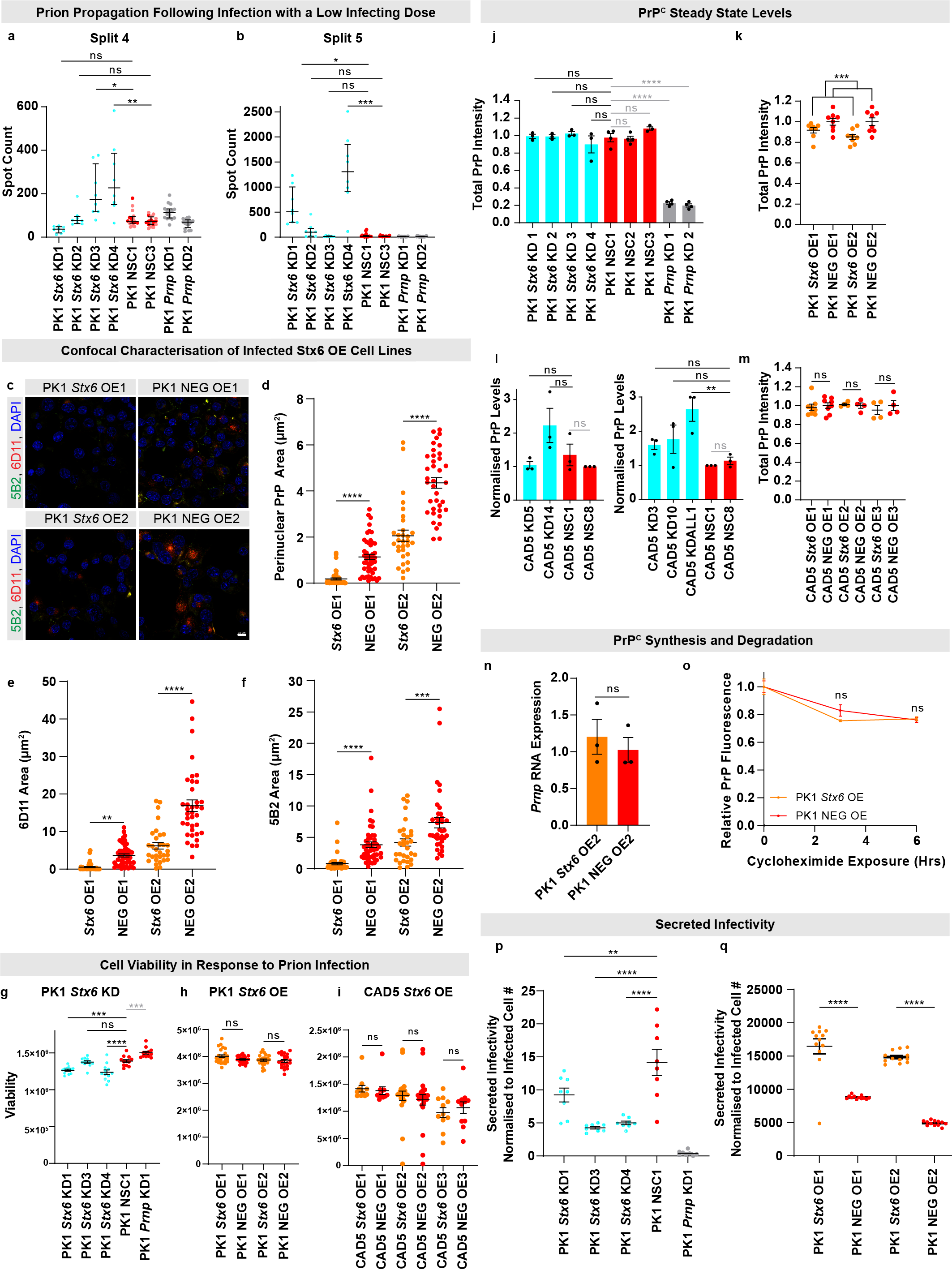


### Supplementary Figure 2 Further Characterisation of PrP-Related Phenotypes and Exploration of Explanators of the Altered Spot Count in Cell Lines with Syntaxin-6 Manipulation. Related to Figure 1. (a,b) Representative example of the spot count of infected cell number at the 4^th^ (a) and 5th (b) split in the SCA following infection of PK1 knockdown cells with a limiting dose of RML prions, 3 x 10^-6^ (8 technical replicates/cell line). As the assay was conducted across multiple plates, spot counts of subsequent plates were normalised to the median spot count of non-silencing control (NSC1) on plate 1. NSC1 was technically replicated across two plates as indicated in separate colours. Significance levels based on Fisher’s exact test of the proportions of wells with a spot count greater than an empirically determined threshold based on the background spot count with uninfected cells. (c) Representative images of two independent *Stx6* overexpression PK1 cell lines and corresponding negative control PK1 cell lines (NEG) stained with the discriminatory anti-PrP antibody pair, 5B2 and 6D11, assessed by laser-scanning microscopy. DAPI, nuclear stain, blue; 6D11, red; 5B2, green. Scale bar, 10 μm. Brightness and contrast of the images were adjusted the same across conditions. (d-f) Quantification of the 6D11 signal area in the perinuclear region (d), total 6D11 staining area (e) and total 5B2 staining area (f) normalised by total cell count as indicated by DAPI. Statistical differences were assessed by one-way ANOVA followed by Fisher’s LSD test on planned comparisons. Each dot represents an individual image (n=31-48/cell line). (g-i) Assessment of cell viability at the 3^rd^ split after prion infection with infected exosomes (n=12 technical replicates/cell line) in PK1 *Stx6* knockdown (g), PK1 *Stx6* overexpression (h), and CAD5 *Stx6* overexpression cell lines (i). Significance levels based on one-way ANOVA with Fisher’s LSD post-hoc test of pre-planned comparisons. (j-m) Assessment of total PrP levels in the different cell lines either by flow cytometry (n=3-7/cell line) (j, k, m) or quantitative western blotting (n=3/cell line) (l). For statistical analysis, an average of the negative control lines was calculated, which was subsequently used to normalize the other values. For PK1 *Stx6* overexpression cell lines, the data was then analysed using a 2-way ANOVA approach, with syntaxin-6 expression level and cell line as the treatment factors. This was followed by pre-planned comparisons of the paired cell lines. For the other cell lines, one-way ANOVA was conducted followed by Fisher’s LSD test. (n) *Prnp* RNA levels as determined by RT-qPCR in one representative paired cell line. Statistical differences were assessed by an unpaired t-test. (o) Cells were treated with 100 µg/mL cycloheximide for the indicated time points (3 replicas/cell line/time point) with total PrP levels subsequently being determined by fluorescence intensity measured by flow cytometry. The initial fluorescence of each cell line was normalised to 1 to monitor the relative protein decay over time. Data represent the mean ± SEM. Statistical differences were assessed by two-way ANOVA followed by Fisher’s LSD test with time and *Stx6* expression as factors, which revealed a significant effect of time (P<0.0001) but not *Stx6* expression. (p,q) Conditioned media was collected from infected PK1 stable *Stx6* knockdown (p) and stable *Stx6* overexpression (q) cell lines and subsequently used to infect PK1 reporter cells followed by SCA analysis. Graphs show the spot count of reporter cells at the 4^th^ split normalised to the spot count of the cells the conditioned media was harvested from to correct for differences in baseline cell-associated infectivity. Statistical differences were assessed by one-way ANOVA followed by Fisher’s LSD test. *P < 0.05, **P < 0.01, ***P < 0.001, ****P< 0.0001.


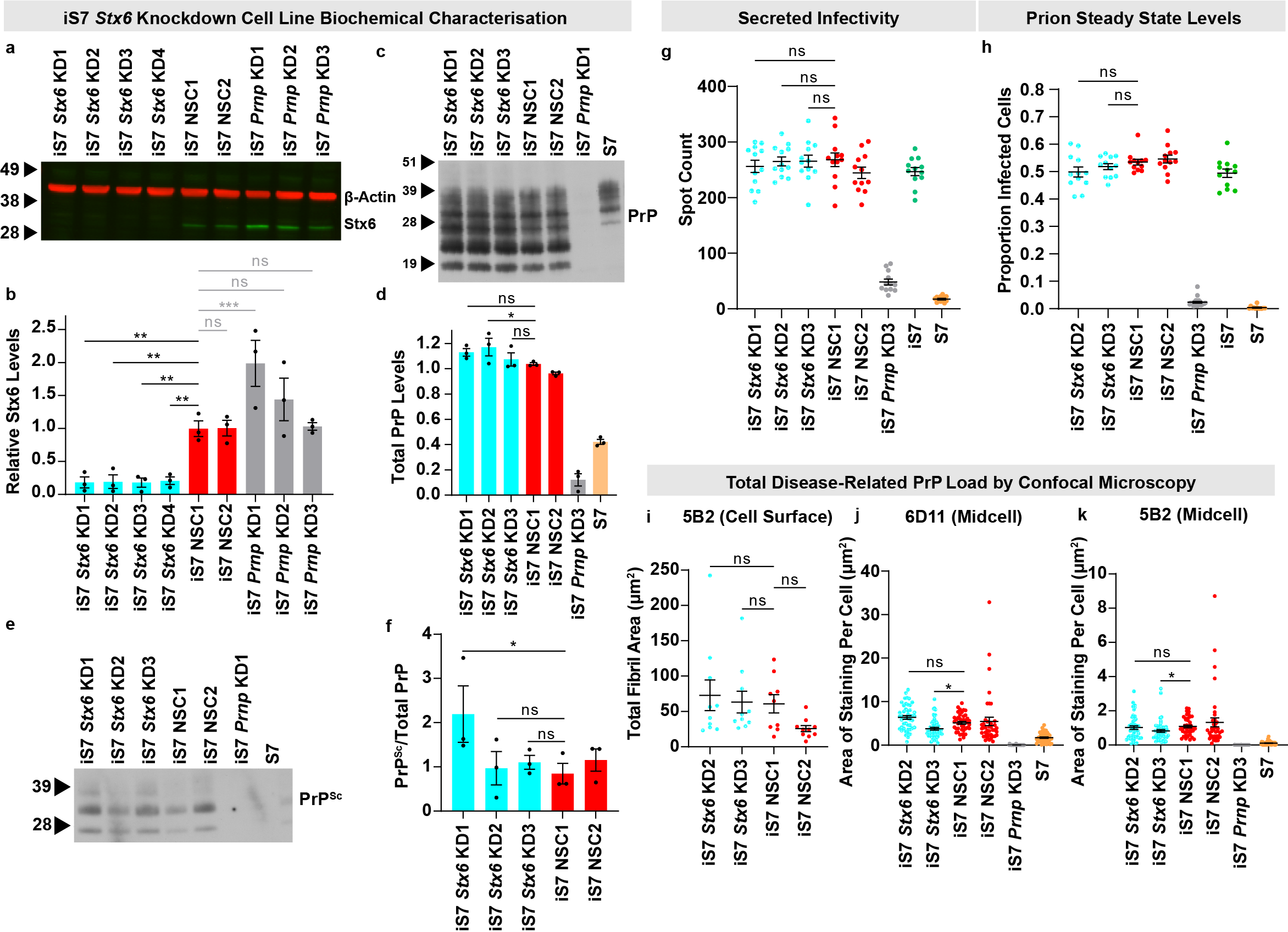


### Supplementary Figure 3 Further Characterisation of Chronically Infected PK1 Cells with Syntaxin-6 Manipulation. Related to Figure 2 and 3. (a) Immunoblot probing for syntaxin-6 levels (bottom, green) and β-Actin (top, red) in chronically infected cells (iS7), which are either stably expressing shRNAs targeting *Stx6* or *Prnp* or shRNAs containing a non-silencing control scrambled sequence (NSC1-2). (b) Quantification of the syntaxin-6 band intensity relative to the β-Actin loading control followed by normalisation to the average normalised signal of the NSC cell lines. Bars show mean ± SEM. Each dot represents a different biological replicate with cells being harvested at three different passage numbers to confirm stable knockdown. Statistical differences were assessed using one-way ANOVA followed by Fisher’s LSD test assessing differences of all cell lines to NSC1. (c) Total PrP load in iS7 cells with *Stx6* manipulation as assessed with immunoblotting with the anti-PrP antibody, 6D11. S7 cells are uninfected cells used as a control. (d) Corresponding quantification with total PrP levels being normalised to an average of the NSC controls with statistics performed as described above (n=3/cell line). (e) Representative immunoblot showing PrP^Sc^ levels following digestion with proteinase K (PK). (f) Quantification of PK-resistant PrP^Sc^ levels, which were first normalised to total PrP levels before normalisation to an average of the NSC controls on each gel with statistics performed as described above. (g) Secreted infectivity was assessed by applying conditioned media from iS7 cell lines with stable *Stx6* manipulation to reporter PK1 cells that were subsequently assessed in the SCA. Graph shows the spot count at split 3 with the line representing the mean ± SEM (individual dots represent 12 technical replicates). Statistical differences were assessed by one-way ANOVA followed by Fisher’s LSD test on planned comparisons. (h) Prion steady state levels in iS7 cell lines were assessed in the SCA. Graph shows the spot count normalised to the hematoxylin total cell count at split 3 to provide a proportion of infected cells. Line represents mean ± SEM (individual dots represent the 12 technical replicates). Statistical differences were assessed by one-way ANOVA followed by Fisher’s LSD test on planned comparisons. (i) Quantification of the total fluorescence area of the elongated disease-related PrP aggregates from the maximum intensity projections at plasma membrane level. Statistical differences were assessed by one-way ANOVA followed by Fisher’s LSD test on planned comparisons (9-10 images/cell line). (j, k) Quantification of total fluorescence area of 6D11 and 5B2 staining normalised to cell number/image by confocal microscopy. Line represents mean ± SEM and individual dots indicating results from a single image (41-49 images/cell line with an average of 43 cells/image). Statistical differences were assessed by one-way ANOVA followed by Fisher’s LSD test on planned comparisons post log transformation. Blots in (C) and (E) were cropped to contain only relevant image data with brightness/contrast optimally adjusted. *p < 0.05, **p < 0.01, ***p<0.001, ****p<0.0001.


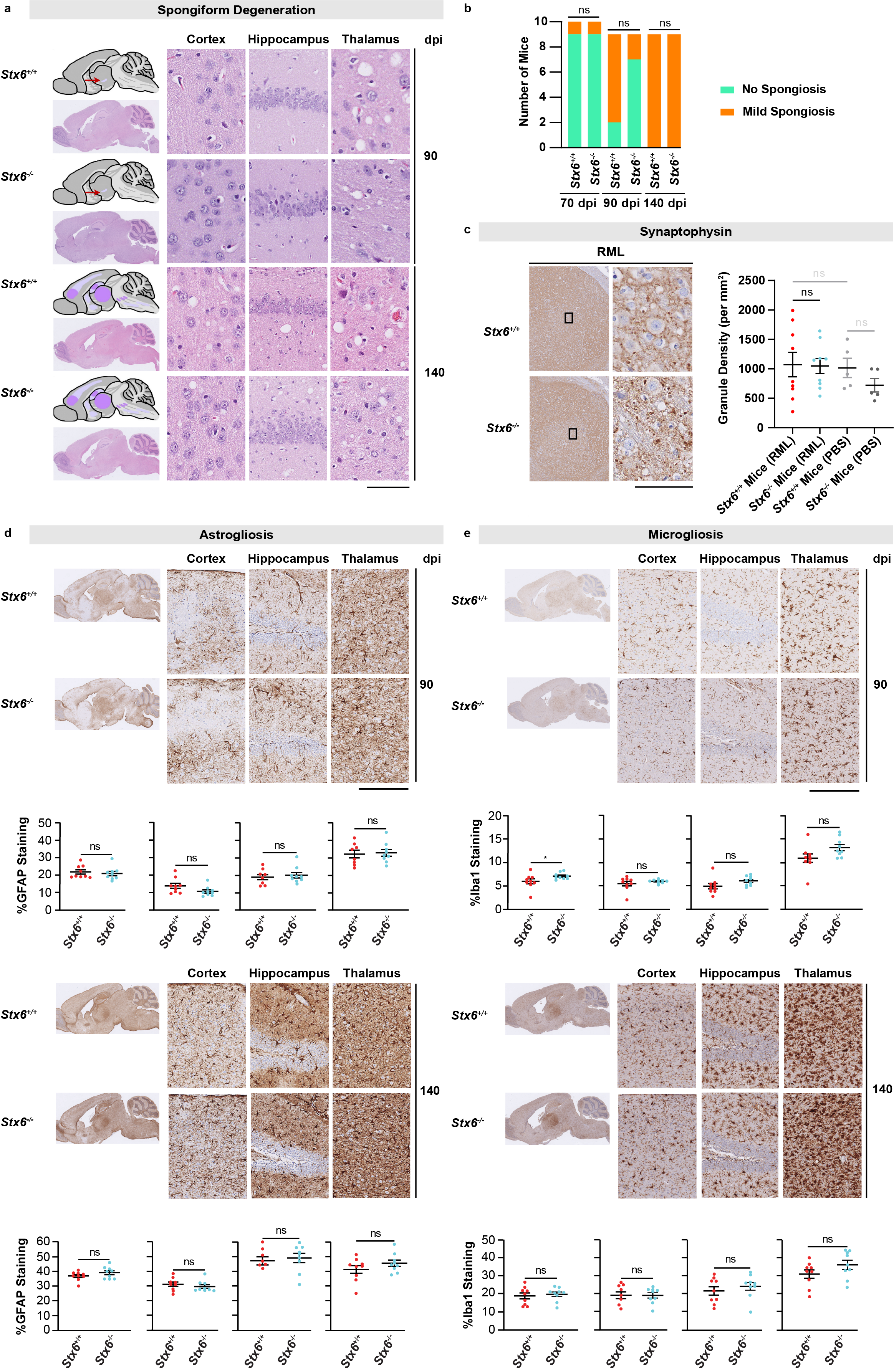


### Supplementary Figure 4 Spatiotemporal Development and Load of the Neuropathological Hallmarks of Prion Disease are Comparable in RML-Infected *Stx6^+/+^* and *Stx6^-/-^* Mice. Related to Figure 5. (a) Spongiform vacuolation was assessed by haematoxylin and eosin (H&E) staining with schematics (left) showing the regional distribution of spongiosis (light lilac, mild spongiosis; medium lilac, moderate spongiosis; dark purple, severe spongiosis) with the red arrow indicating the mild spongiosis seen in the inferior thalamus at 90 days post inoculation (dpi). Representative images of whole brain sections are shown below the schematics as well as the cortex, hippocampus and thalamus at 90 and 140 dpi (right). Scale bar, 60 μm (cortex and thalamus), 120 μm (hippocampus), 1.5 mm (whole section). (b) Scoring of spongiosis with statistical differences at each time point assessed using Fisher’s exact test. (c) Overview of synaptophysin staining in the thalamus in RML-inoculated *Stx6^+/+^* and *Stx6^-/-^* mice. The square indicates the approximate area corresponding to the high magnification image. Scale bar, 1 mm (left), 50 μm (right). Quantification of synaptic granule density in the thalamus at 140 dpi is shown on the right. Statistical differences were assessed using one-way ANOVA with the planned comparison between RML-infected *Stx6^+/+^* and *Stx6^-/-^* mice. (d) Astrogliosis was assessed by quantification of GFAP staining at 90 dpi and 140 dpi in neuropathologically validated infected animals. Representative images are shown with the quantification indicated below. Post-rank transformation, a 2-way repeated measures mixed model approach was used for statistical analysis using the unstructured covariance structure to model the within-subject correlations, with genotype as the treatment factor and brain region as the repeated factor. This was followed by planned comparisons on the predicted means to compare the effect genotype on staining in the different brain regions. Scale bar, 250 μm (cortex, hippocampus, thalamus), 2.5 mm (whole section). (e) Microgliosis was assessed by quantification of Iba1 staining at 90 dpi and 140 dpi in neuropathologically validated infected animals. Representative images are shown with the quantification indicated below. Post-rank transformation, a 2-way repeated measures mixed model approach was used for statistical analysis using the unstructured covariance structure to model the within-subject correlations, with genotype as the treatment factor and brain region as the repeated factor. This was followed by planned comparisons on the predicted means to compare the effect of genotype on staining in the different brain regions. Scale bar, 250 μm (cortex, hippocampus, thalamus), 2.5 mm (whole section).


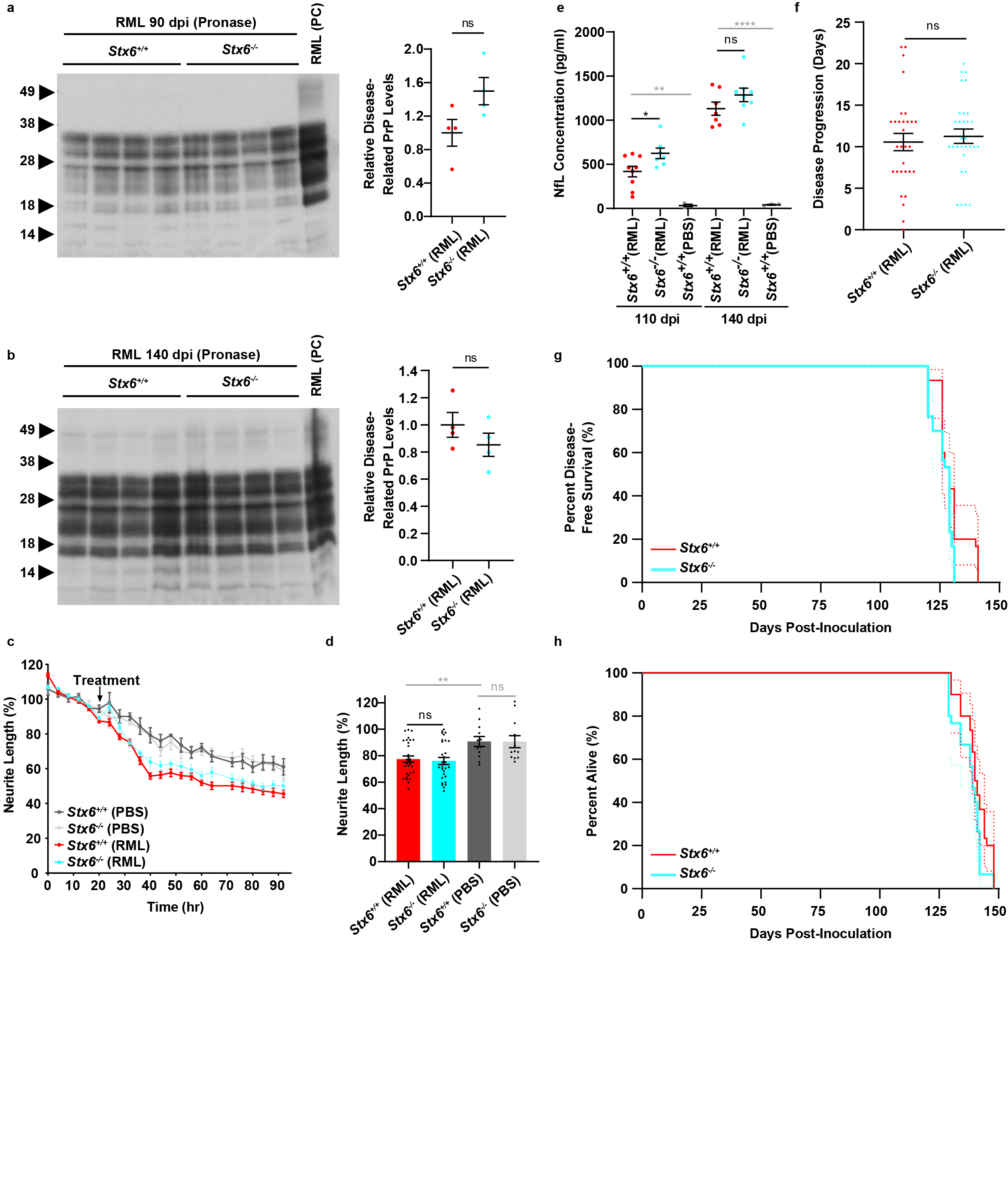


### Supplementary Figure 5 No Differences in Neurotoxicity-Related Outcome Measures in RML-Infected *Stx6^+/+^* and *Stx6^-/-^* Mice. Related to Figure 5. (a) Immunoblotting with the anti-PrP antibody, ICSM35, of brain homogenates from mice 90 days post inoculation (dpi), following pronase E digestion (100 µg/ml, 37°C, 30 min). The corresponding semi-quantification of the disease-related PrP signal is shown on the right. Each sample was normalised to the average of RML-infected *Stx6^+/+^* mice with statistical differences assessed using the Student’s t-test. Bar graphs represent mean ± SEM of 4 biological replicates/genotype. PC, positive control. (b) Pronase digested brain homogenates at 140 dpi. Brightness and contrast of the western blots were optimally adjusted. (c) Representative neurotoxicity assay showing normalised neurite retraction over time in primary neurons in response to treatment with a 2.5 x 10^-4^ concentration of RML-infected *Stx6^+/+^* and *Stx6^-/-^* brain homogenates from mice culled at 140 dpi (n=10 technical replicates). PBS-inoculated *Stx6^+/+^* and *Stx6^-/-^* controls culled at 140 dpi were also analysed in quadruplicate. Graph shows mean ± SEM. (d) Neurite retraction 12 hrs post-treatment in 3 independent cell cultures with statistical differences assessed using one-way ANOVA followed by Fisher’s LSD post-hoc test. (e) Serum NfL levels in RML-infected *Stx6^+/+^* and *Stx6^-/-^* mice at 110 dpi (8-9/genotype) and 140 dpi (7-9/genotype) were measured. PBS-inoculated *Stx6^+/+^* mice (n=5/time point) were also included as controls. Graphs show mean ± SEM, with symbols representing individual animals. Data were analysed using one-way ANOVA followed by Fisher’s LSD post-hoc test. (f) Graph showing disease progression, defined as the difference between time to first symptom to prion disease diagnosis (days). P-value derived from an unpaired t-test. (g) Kaplan Meier plot showing disease-free survival probability until animals developed first symptoms of prion disease. Dashed lines represent 95% confidence intervals. (h) Kaplan Meier plot showing incubation periods (time from inoculation to definite prion disease diagnosis). *P < 0.05, **P < 0.01, ****P < 0.0001.

# Supplementary Tables

### Supplementary Table 1. Transcriptomic Analysis of Uninfected *Stx6*^+/+^ and *Stx6*^-/-^ Mouse Brain to Explore Compensatory Mechanisms. Table showing the top 50 STRING interactions for syntaxin-6 and the average normalised read counts for *Stx6*^+/+^ (WT, n=4) and *Stx6*^-/-^ (KO, n=5) mice, as well as the associated fold changes and p-values.

Excel file uploaded separately.
